# Compensatory eating after exercise in everyday life: Insights from daily diary studies

**DOI:** 10.1371/journal.pone.0282501

**Published:** 2023-03-15

**Authors:** Natalie M. Reily, Rebecca T. Pinkus, Lenny R. Vartanian, Kate Faasse

**Affiliations:** 1 Black Dog Institute, Randwick, NSW, Australia; 2 School of Psychology, The University of Sydney, Sydney, NSW, Australia; 3 School of Psychology, UNSW Sydney, Sydney, NSW, Australia; Bangor University, UNITED KINGDOM

## Abstract

There is considerable variability in how successful people are in losing weight via exercise programs. Experimental research suggests that greater food intake after exercise may be one factor underlying this variability, but no studies have assessed patterns of post-exercise eating behaviour over time in naturalistic settings. Thus, we aimed to assess how exercise and contextual factors (e.g., hunger, presence of others) influence the healthiness and amount of food eaten after exercise in two daily diary studies. In Study 1, participants (*n* = 48) reported their food intake and exercise daily for 28 days. For each meal, they provided a brief description of the food(s) eaten which were then categorised as healthy, unhealthy, or mixed (neither healthy nor unhealthy) by two independent coders. Study 2 used the same method, but participants (*n* = 55) also reported the portion size of each meal. Hierarchical linear modelling showed that in Study 1, contrary to expectations, post-exercise meals were less likely to be unhealthy (relative to mixed) than were random meals from non-exercise days (OR = 0.63, *p* = .011), and that participants ate proportionally fewer unhealthy meals on exercise days compared to non-exercise days (*b* = -4.27, *p* = .004). Study 2 replicated these findings, and also found that participants consumed larger meals after exercise in comparison to random meals from non-exercise days (*b* = 0.25, *p* < .001). Participants were not consistently engaging in compensatory eating by eating less healthily after exercise compared to on non-exercise days, but they did eat larger portions post-exercise. This work highlights the need for naturalistic methods of assessing compensatory eating, and has the potential to facilitate development of strategies to improve health behaviour regulation.

## Introduction

Globally, 42% of adults are actively trying to lose weight, and an additional 23% are trying to maintain their current weight [1]. Among the most common strategies used for weight control are monitoring dietary intake and engaging in physical activity and exercise [2]. Despite these strategies being frequently used, the average amount of weight people lose is modest, and the weight loss is difficult to sustain over time [3–5]. A meta-analysis estimated weight loss with diet interventions or diet and exercise interventions to be only 1.6 kg on average [6]. One possible reason for why weight control attempts are often unsuccessful is that people who start exercising might also compensate for that exercise by engaging in other behaviours that make losing weight more difficult. Specifically, people may alter their eating habits in response to exercise by increasing their food intake or eating less healthily after having exercised [7–9]. This compensatory eating behaviour might underlie some of the variability observed in the amount of weight loss in response to exercise [3, 5], and undermine fitness, health and weight goals.

Many studies have investigated compensatory eating after exercise in laboratory settings. A robust meta-analysis of laboratory studies showed that exercise led to a slight but nonsignificant increase in subsequent energy intake compared to intake after a control session not involving exercise [10]. In contrast to these studies, other studies have demonstrated effects of compensatory eating after exercise by influencing participants’ perceptions of exercise [11–14]. For example, one study had participants exercise on a stationary bicycle until they had burned 120 kcal, but participants were falsely informed that they had either burned 50 kcal or 265 kcal [13]. Participants who were told that they had burned 265 kcal consumed more food in a subsequent taste test compared to those who were told that they had burned 50 kcal. Another study had participants complete one of three 5 min tasks: exercising (by doing step-ups), imagining exercising (imagining walking up stairs), or imagining attending a classical music concert (no exercise; [12]). Intake for participants in the actual exercise condition and the no-exercise control group was not significantly different, but those that imagined exercising consumed fewer calories than the other two groups.

Other studies using within-subjects designs have also predominately found no group-level differences in consumption after exercise compared to consumption after rest [15, 16]. For example, in one study, participant completed two laboratory sessions, one-week apart [16]. The two sessions involved cycling on a stationary bicycle for 50 min (exercise condition) or reading quietly for 50 min (no-exercise condition) and then eating lunch in the laboratory. There was no difference in overall intake between conditions, but there was considerable variability in participants’ eating behaviour after they had exercised compared to when they had not exercise. Specifically, about 50% of participants were classified as “compensators” because they consumed more food after exercise compared to no exercise, whereas the rest of the participants were classified as “non-compensators” because they either did not alter their food intake or they ate less after exercise compared to after no exercise. Other studies have also found variability in eating behaviour after exercise compared to rest, ranging from up to approximately 250 kcal *more* after exercise than rest for some studies, and up to approximately 200 kcal *less* after exercise than rest for other studies [17, 18].

An additional complexity is that people’s patterns of intake after exercise may not be consistent across exercise sessions [19, 20]. For example, Unick et al. (2015) measured participants’ food intake after exercise and after rest in a within-subjects design, but participants completed three pairs of testing sessions, each separated by one week [20]. Importantly, they identified that the dichotomous classification of people as compensators and non-compensators was not stable over time, with only 21% of their sample consistently classified across all three pairs of testing sessions. Given that most studies of compensatory eating have examined eating behaviour after one or a few exercise sessions at most, the frequency of compensatory eating over time remains unknown.

One promising cluster of methodologies that can be used to assess compensatory behaviour over an extended time periods is “daily life methods”, which assess the occurrence of particular behaviours, events, or experiences in naturalistic settings [21]. Sampling can vary in frequency from once per day (daily diary studies; [22]) to several times a day (ecological momentary assessment, experience sampling; [23, 24]), and in duration from a few days to weeks or months, depending on the estimated frequency of the behaviour of interest [25].

Daily life methods are a more ecologically-valid method because they allow participants to report on experiences in their typical environment, rather than in a laboratory scenario. In most compensatory eating laboratory experiments, participants are served a limited range of foods, which is in stark contrast to the modern food environment in which food is often varied, saliently advertised, and ubiquitously available. Similarly, in everyday life, people can choose from a variety of exercise activities rather than being prescribed a specific mode of exercise. Laboratory studies of compensatory eating therefore might be problematic because having choice around parameters of the exercise leads to lower subsequent food intake in comparison to no choice [26]. To date, only one study has explored compensatory eating in free-living conditions [27]. In that study, participants took part in an 8-week walking intervention, with food intake measured via 24-hour dietary recall on one exercise day and one non-exercise day. Food intake was greater in the three hours after exercise, indicative of compensatory eating, although there was no difference in total energy intake on the exercise day compared to the non-exercise day. These results highlight the need for further work to examine compensatory eating in naturalistic settings.

Daily life methods can also assess the frequency of particular events because of their ability to track people over extended time periods [25]. This kind of repeated measurement approach can provide insight into individual variability in patterns of post-exercise eating behaviour, which remains a blind spot in the current literature due to a focus on consumption after a single exercise session. Further, daily life methods lend themselves to a comprehensive exploration of the parameters around post-exercise eating behaviour. For example, contextual factors such as social cues [28] and other external cues such as eating location, food availability, and portion size [29] have been shown to influence eating behaviour. It is possible that people may be more likely to engage in post-exercise compensatory eating under certain contextual conditions, and an extended daily diary approach would allow for assessment of the contextual effects.

Two recent daily life methods studies provide some information about the relationship between exercise and eating behaviour [30, 31], even though neither was specifically designed to examine compensatory eating after exercise. Dohle and Hofmann (2019) asked participants to complete five short surveys per day for one week about their recent health behaviours, with the aim of examining the relationships among healthy and unhealthy behaviours [30]. One notable finding was that, when participants indicated that unhealthy eating was linked to a previous healthy behaviour, that previous healthy behaviour was most often exercise or healthy eating. However, a limitation of this study was that the contingencies between behaviours that were captured were based on participants’ subjective self-reports of the relationships between their current behaviour and a previous behaviour. Therefore, any additional patterns of behaviour that participants themselves were not aware of might not have been detected. Another study by Grenard et al. (2013) found that having exercised for at least 60 min on a particular day was associated with increased likelihood of consuming sweetened beverages but was unrelated to sweet and salty snack consumption [31]. Note, however, that those researchers did not specifically examine food consumption immediately after exercise. Overall, these two studies suggest that daily life methods could be a useful approach to exploring post-exercise compensatory eating.

The primary aim of the current research was to use a daily diary approach to test whether people engage in compensatory eating after exercise in everyday life. In Study 1, we compared the healthiness of meals on exercise days to non-exercise days, and the healthiness of post-exercise meals to random meals drawn from non-exercise days. Study 2 aimed to replicate the findings of Study 1, as well as to examine the size of meals eaten on exercise days compared to non-exercise days, and the size of post-exercise meals compared to random meals. A secondary aim of both studies was to explore whether any contextual factors (e.g., food availability, feeling hungry) or characteristics of exercise (e.g., type, duration and intensity) influenced the healthiness (Study 1 and 2) and portion size (Study 2) of post-exercise meals.

## Study 1

Participants reported their food intake and exercise behaviour at the end of each day for 28 days. Given that it was important to capture multiple instances of both exercise and compensatory eating, a reasonably long duration of 28 days was used. Once-per-day sampling was chosen over sampling multiple times per day to minimise participant burden and ensure good compliance over this longer duration [22]. Previous laboratory research has produced mixed findings for compensatory eating, and there is evidence of variability in post-exercise eating behaviour (e.g., [16, 17]) suggesting that people may only compensate some of the time. Thus, although we hypothesised that, across days, participants would eat more unhealthily on exercise days compared to non-exercise days on average, we also expected some within- and between-person variability. Regarding the secondary aim, given the lack of prior research in the area, we did not have any specific hypotheses about the influence of contextual factors.

### Method

#### Participants

Participants were Australian community members (*n* = 61) who were recruited via online advertisements. Sample sizes of at least 30 participants are recommended for daily diary studies with survey days nested within participants [32, 33]. To be eligible to participate, participants had to be over 18 years old and have a mobile phone with internet access. They also had to have exercised between 8–20 times in the last 28 days (i.e., approximately 2–5 times per week). Moderate exercisers were selected given that infrequent exercisers (0–1 days per week) were unlikely to provide sufficient exercise sessions across the survey period, and frequent exercisers (6–7 days per week) were unlikely to provide sufficient non-exercise days across the survey period. Participants also had to report that they had eaten less healthily after at least 30% of their reported exercise sessions (36.7% of potential participants were deemed ineligible for failing to meet this threshold). This requirement was put in place to ensure that sufficient opportunities for compensatory eating were captured across the 28-day study period. Participants were required to complete at least 20 surveys to receive full reimbursement, and those who completed fewer than 18 surveys (*n* = 13) were excluded, leaving a final sample of 48 participants (34 women, 14 men). The mean age of the sample was 28.71 years (*SD* = 10.05; range = 18–59). The mean body mass index (BMI; kg/m^2^) was 24.34 (*SD =* 3.95; range = 17.58–39.18). Regarding ethnicity, 44% identified as White, 44% identified as Asian, 2% identified as Aboriginal/Pacific Islander, and 10% identified as “other”. Most participants (75%) wanted to lose weight, and 60% were currently dieting or watching what they ate.

#### Daily surveys

The daily surveys were modeled on dietary assessment tools which require participants to systematically report their meals in sequential order [34, 35]. Many of these tools are as accurate as traditional prompted 24-hour recall via phone or interview [36], but they are also time consuming to complete. In order to encourage participant compliance and avoid undue participant burden, we used a briefer measure that allowed us to capture multiple instances of compensatory eating with less precision (rather than a few instances with high precision). At the end of each day, participants reported their food intake for the day by entering their meals one-by-one in the order that they ate them. For each eating occasion, they specified the type of meal (main meal or snack), a brief description of the food(s) eaten, and time of the eating occasions (to the nearest 30 min). Descriptions of food(s) eaten were coded for healthiness by the research team (details below). Next, participants were asked whether or not they had exercised on that day. Participants were informed that exercise in the context of the study referred to structured and purposeful physical activity (rather than incidental physical activity). If they had exercised, they were then asked to specify the exercise type (aerobic/cardio, strength, balance/flexibility, sport, or combination of these options), intensity (vigorous, moderate, or low), start time (to the nearest 30 min) and duration (in 30-min increments). Participants were then given the option to add additional exercise sessions if needed.

Next, participants were asked follow-up questions about one target eating occasion from that day. If they had exercised, these questions were about the first eating occasion after the first exercise session they had reported. If participants had not eaten after exercising, or had not exercised that day, they were asked about a randomly selected eating occasion. For the target eating occasion, participants were asked how healthy the eating occasion was (more healthy than normal, the same as normal, less healthy than normal). They then answered 11 yes/no “context” questions about the circumstances surrounding the meal. Specifically, participants indicated whether or not they were: feeling hungry, feeling stressed, in a bad mood, feeling tired, eating alone, in a rush, eating at home, planning to eat the food, or experiencing cravings for that food; and whether or not they had other food options, and whether or not the food was readily available. Finally, participants were shown a list of all their eating occasions from that day. They were asked to rate each meal they had consumed in terms of whether it was less healthy than normal, the same as normal, or more healthy than normal.

#### Procedure

After providing written informed consent, participants completed demographic information in an initial questionnaire, including age, gender, height and weight (used to calculate BMI), and ethnicity. They were also asked whether or not they were dieting, and whether they wanted to lose weight, stay the same weight, or gain weight. Participants also read instructions about the end-of-day surveys and provided their usual bedtimes. After completing this questionnaire, participants were contacted by the researcher to confirm their end-of-day survey start date and mobile phone number and to provide an opportunity for participants to ask any questions. Each night (1 hr before their nominated bedtime), participants were sent SMS messages containing the end-of-day survey link, which they completed on their mobile phones (95% of surveys were completed on time, i.e., the same evening). At the end of the 28-day period, participants were sent debriefing and recompense information. Participants were compensated a maximum of AUD $70 if they completed the initial questionnaire and at least 5 surveys in all 4 weeks of the study. The study protocol was approved by UNSW Sydney’s Human Research Ethics Advisory Panel (HC3107).

#### Coding of meal healthiness

The research team coded the descriptions that participants provided about the foods they had eaten in terms of how healthy each eating occasion was (note that the terms “eating occasion” and “meal” are used interchangeably in this paper). Meal descriptions were coded into four categories (healthy, unhealthy, mixed, and “exclude”) using the most recent Australian Guide to Healthy Eating [37]. Healthy meals were those that consisted of foods from the five recommended food groups: grains, lean meats, reduced-fat diary, fruit, and vegetables (e.g., “wholegrain toast with poached eggs and avocado”). Unhealthy meals consisted of foods from the discretionary foods section of the guide, which included alcohol, high sugar or high fat products, and fast food (e.g., “chocolate choc chip muffin”). Mixed meals were those that were neither clearly healthy nor clearly unhealthy (e.g., “sandwich”), and also included meals that consisted of both healthy and unhealthy food items (e.g., “stir fry with vegetables and brown rice, ice cream”). Finally, meals were excluded from analyses if they consisted only of items with zero or negligible calories (e.g., “water” or “herbal tea”) or if the food could not be identified due to typographic error (e.g., “lentens”; *n* = 14; post-exercise meals = 1; random meals = 13).

Two coders independently coded a random subset of 20% of the total surveys (1,030 meals). Cohen’s kappa for this initial subset was .52, indicating weak inter-rater agreement [38]. The coders discussed discrepancies between meal classifications and resolved them with the input of a third researcher. Some systematic differences in coding were identified (e.g., zero calorie beverages, unfamiliar foods) that appeared to account for a substantial proportion of the disagreement. After resolving discrepancies and refining the coding scheme, the two coders coded a second subset of 10% of the total surveys (511 meals). Cohen’s kappa for the second subset was .81, indicating strong inter-rater agreement. Discrepancies were again resolved with a third researcher. Given the substantial agreement in the second subset, Coder 1 then coded the remaining surveys using the refined coding scheme.

#### Statistical analysis

Preliminary descriptive analyses pertaining to the frequency of eating and exercise across the 28 days were computed using SPSS 25. Due to the multilevel structure of the data (daily surveys nested within participants), the primary analyses were carried out using the multilevel modeling software package HLM7 [39]. In these analyses, variables from the end-of-day surveys (exercise vs. non-exercise days, post-exercise vs. random meals, contextual factors) are Level-1 variables, whereas variables pertaining to participant characteristics (individual differences) are Level-2 variables. Models with categorical outcomes were analysed using hierarchical generalised linear modelling (HGLM), and models with continuous outcomes were analysed using hierarchical linear modelling (HLM).

The primary analyses examined the healthiness of eating occasions based on codes (unhealthy, mixed, or healthy) derived from participants’ descriptions of the foods they ate. Meal healthiness as coded from participants’ meal descriptions was significantly correlated with their self-reports of meal healthiness, *r* = .49, *p* < .001. The coded measure of healthiness was used for the analysis as a relatively more objective assessment of the foods consumed given that participants’ subjective ratings of relative meal healthiness are more likely to be inaccurate or influenced by demand characteristics or reporting biases.

Compensatory eating was examined in two different ways. First, compensatory eating was explored *at the meal level* by comparing the healthiness of the subset of post-exercise meals (*n =* 437 meals) to a subset of randomly-selected meals drawn from non-exercise days (*n =* 593 meals). Second, compensatory eating was assessed *at the day level* in the full dataset by testing whether exercise (yes/no) was a predictor of the percentage of meals on a particular day that were unhealthy. As an additional analysis, we also tested whether exercise predicted the number of main meals consumed per day and the number of snacks consumed per day.

Regarding the secondary aim, the post-exercise meal subset was examined to determine whether the healthiness of post-exercise meals was predicted by any contextual factors or characteristics of the exercise. The same analyses were also carried out in the non-exercise day random meal subset to determine whether the same contextual factors predicted healthiness of meals on non-exercise days as for post-exercise meals.

### Results

#### Descriptive findings

The mean number of surveys completed per participant was 25.25 surveys (*SD* = 2.44). There were a total of 1,214 recorded end-of-day surveys capturing 5,115 total eating occasions. On average, participants ate 2.62 main meals (i.e., breakfast, lunch, or dinner; *SD =* 0.37) and 1.55 snacks (*SD* = 1.13) per day.

*Exercise sessions*. There were 608 total days on which participants reported exercising, and participants exercised an average of 12.67 days during the 28-day survey period (*SD* = 4.80 days). Participants predominately reported engaging in only one exercise session per day (*n =* 514), although there were some days on which participants completed two sessions (*n* = 85) or more than two sessions (*n* = 8). The analyses focused on the first exercise occasion, of which 346 sessions were described as aerobic/cardio, 87 were strength, 53 were balance/flexibility, 27 were sport, and 94 were a combination. The modal duration of these sessions was 30–60 min. Regarding intensity of exercise, 22.73% of sessions were vigorous, 47.12% were moderate, and 30.15% were low intensity.

*Meals*. There were 438 eating occasions that followed an exercise session (there were no post-exercise meals on 170 days). Of the post-exercise meals that could be coded for healthiness (*n* = 437), 25.86% were unhealthy, 40.73% were mixed, and 33.41% were healthy. One random meal was captured from each of the non-exercise days (*n* = 606) to be compared to the post-exercise meal subset. Of those meals that could be coded for healthiness (*n =* 593), 37.61% were unhealthy, 36.76% were mixed, and 25.63% were healthy.

**Fig 1:**
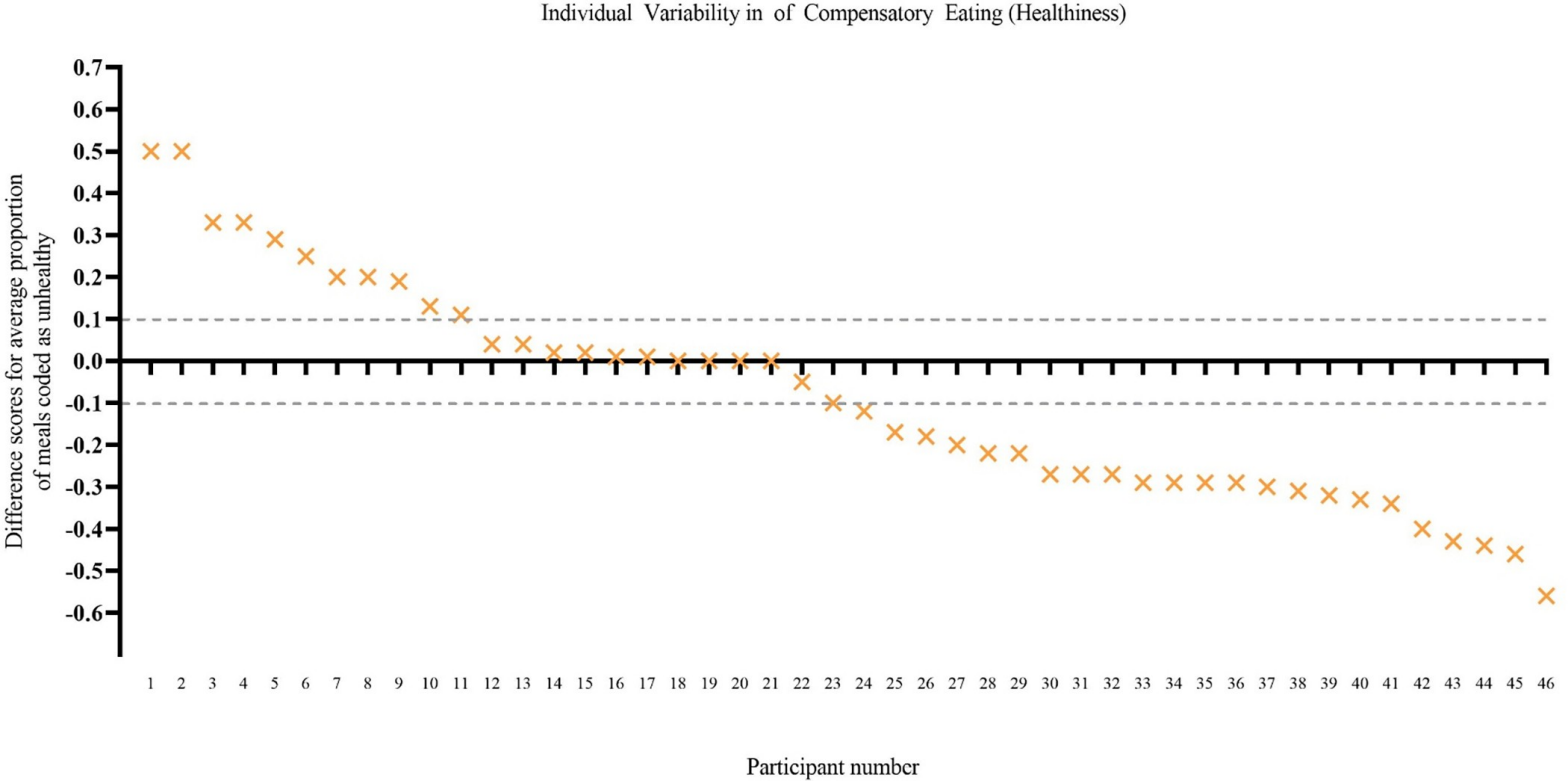
Individual variability in patterns of eating (Study 1). The y axis shows a difference score for each participant: the proportion of post-exercise meals that were unhealthy minus the proportion of random meals that were unhealthy. A positive score indicates that, on average, the participant consumed a greater proportion of unhealthy meals post exercise than at random meals (i.e., compensatory eating). Dotted lines indicate the boundaries (± 0.10) used to mark a pattern of eating that was indifferent to exercise (similar proportion of unhealthy meals after exercise and at random meals). Participant scores are ordered in descending order from most compensatory eating to least compensatory eating. Difference scores could only be calculated for 46 of the 48 participants because two did not have any post-exercise meals.

*Individual variability*. There was considerable individual variability in patterns of eating behaviour. When comparing the proportion of post-exercise meals that were coded as unhealthy to the proportion of random meals on non-exercise days that were coded as unhealthy for each participant (averaging across days), 11 participants showed a pattern of compensatory eating behaviour (greater proportion of unhealthy meals after exercise compared to random meals on non-exercise days), 24 showed the opposite pattern (smaller proportion of unhealthy meals after exercise compared to random meals), and 11 showed eating behaviour that was indifferent to exercise (similar proportions—no more than 10% difference—in unhealthy post-exercise meals and unhealthy non-exercise day random meals; see Fig 1).

#### Exercise and healthiness of meals

The primary outcome variable of interest was the healthiness of eating occasions (unhealthy, healthy, and mixed). Mixed was used as the reference category, such that results describe the relative likelihood that eating occasions were unhealthy compared to mixed and the relative likelihood that eating occasions were healthy compared to mixed. Relative likelihoods are described in the following results as odds ratios. To simplify the presentation, comparisons between unhealthy and mixed will be referred to as “eating unhealthily” and comparisons between healthy and mixed will be referred to as “eating healthily.” First, to determine whether there was any evidence of compensatory eating at the meal level, the healthiness of meals was compared for post-exercise eating occasions and random eating occasions drawn from non-exercise days. Exercise (1 = post-exercise eating occasion, 0 = random eating occasion on non-exercise day) was a significant predictor of eating unhealthily such that, contrary to expectations, participants were relatively *less* likely to eat unhealthily at post-exercise eating occasions (predicted odds = 0.60) compared to random eating occasions on non-exercise days (predicted odds = 0.96). Exercise was not a significant predictor of eating healthily (see Table 1).

**Table 1:** Exercise as a predictor of meal healthiness (Study 1).

		Odds ratio	Lower limit	Upper limit	*b*	*SE*	*t*	*p*
Unhealthy vs. mixed meals								
	Intercept^a^	0.96	0.74	1.25	-0.04	0.13	-0.31	.757
	Post-exercise eating occasion	**0.63** ^**b**^	**0.44**	**0.90**	**-0.46**	**0.18**	**-2.54**	**.011**
Healthy vs. mixed meals								
	Intercept^a^	0.63	0.46	0.85	-0.47	0.15	-3.11	.003
	Post-exercise eating occasion	1.10	0.78	1.55	0.09	0.18	0.53	.597

*Note*. Meal healthiness was a multicategorical outcome variable with three categories (unhealthy, mixed, healthy), generating two comparisons against mixed, the reference category: (1) Unhealthy vs. mixed meals and (2) Healthy vs. mixed meals. Exercise was a dichotomous predictor (1 = post-exercise eating occasion, 0 = random eating occasion on non-exercise day). Bold denotes that the predictor was significant.

^a^Intercept of the hierarchical generalized linear model (HGLM) refers to when the value of both the exercise predictor = 0 (i.e., random eating occasion on non-exercise day) and meal healthiness = 0 (i.e., mixed, reference category).

^b^For ease of interpretation of the results, the odds ratio for eating unhealthily (likelihood of eating an unhealthy meal compared to a mixed meal) has been parsed into two predicted odds values, which are expressed in the text. Predicted values in the text are derived from $e^{\left(b_{0}+bx\right)}$ substituting in the values of the predictor, *x*.

Second, compensatory eating was examined at the day level by assessing whether exercise was a predictor of the percentage of eating occasions within a day that were unhealthy. Exercise (1 = yes, 0 = no) was a significant predictor of the percentage of unhealthy eating occasions, such that participants ate proportionally fewer unhealthy meals on exercise days (predicted value = 30.68%) than on non-exercise days (predicted value = 34.95%; see Table 2).

**Table 2:** Exercise as a predictor of the percentage of unhealthy meals in a day (Study 1).

	*b*	*SE*	*t*	*p*
Intercept^a^	34.95	2.56	13.66	< .001
Exercise	**-4.27** ^b^	**1.43**	**-2.99**	**.004**

*Note*. Percentage of eating occasions that day coded as unhealthy was a continuous outcome (0%–100%). Exercise was a dichotomous predictor (1 = yes, 0 = no). Bold denotes that the predictor was significant.

^a^Intercept of the hierarchical linear model (HLM) refers to when the exercise = 0 (non-exercise day).

^b^Predicted values in the text are derived from: *b*_*0*_ + *bx*, substituting in the values of the predictor, *x*.

#### Exercise and frequency of meals

Exercise was a significant predictor of the number of main meals consumed such that participants ate relatively more main meals on exercise days (predicted event rate = 2.72 main meals) compared to non-exercise days (predicted event rate = 2.55 main meals). However, exercise was not a significant predictor of the number of snacks (see Table 3).

**Table 3:** Exercise as predictor of number of main meals and snacks consumed (Study 1).

		Event rate ratio	Lower limit	Upper limit	*b*	*SE*	*t*	*p*
Main meals								
	Intercept^a^	2.55	2.46	2.64	0.94	0.02	51.94	< .001
	Exercise	**1.07** ^b^	**1.01**	**1.12**	**0.06**	**0.03**	**3.09**	**.002**
Snacks								
	Intercept^a^	1.54	1.47	1.61	0.43	0.12	3.65	< .001
	Exercise	1.06	1.00	1.13	0.06	0.07	0.84	.404

*Note*. Main meals was a count variable of the number of main meals (breakfast, lunch, dinner) eaten in one day. Snacks was a count variable of the number of snacks eaten in one day. Exercise was a dichotomous predictor (1 = yes, 0 = no). Bold denotes that the predictor was significant.

^a^Intercept of the HGLM refers to when the value of the exercise predictor = 0 (non-exercise days).

^b^Predicted values in the text are derived from $e^{\left(b_{0}+bx\right)}$ substituting in the values of the predictor, *x*.

#### Predictors of post-exercise meal healthiness

To explore post-exercise eating behaviour further, contextual factors and characteristics of exercise were examined as predictors of meal healthiness in the post-exercise meal subset (*n* = 437).

*Contextual predictors*. The 11 contextual factors referring to parameters of the meal were tested as predictors of post-exercise eating occasion healthiness for the subset of post-exercise eating occasions. Predictors were first examined individually, and then any significant predictors were entered simultaneously into an overall model. Given the exploratory nature of these analyses, no adjustment was made for multiple comparisons, and therefore these results should be interpreted with caution.

There were five significant individual predictors: Participants were relatively more likely to eat unhealthily when they were not hungry (predicted odds = 1.04) compared to when they were hungry (predicted odds = 0.47). Participants were also relatively more likely to eat unhealthily when they were in a bad mood (predicted odds = 2.34) than when they were not in a bad mood (predicted odds = 0.52). Unplanned meals were relatively more likely to be unhealthy (predicted odds = 1.10) than were planned meals (predicted odds = 0.29), and participants were also more likely to choose unhealthy options at post-exercise meals when they had to go out of their way to obtain the food (predicted odds = 0.98) compared to when it was readily available (predicted odds = 0.49). Finally, participants were more likely to eat unhealthily after exercise when they were not at home (predicted odds = 0.87) compared to when they were at home (predicted odds = 0.39).

There were only two significant predictors of eating healthily (relative to mixed) after exercise. Participants were more likely to eat healthily when there were no other food options (predicted odds = 0.74) compared to when other food options were available (predicted odds = 0.38). Post-exercise meals were also more likely to be healthy when participants felt they were not in a rush (predicted odds = 0.75) compared to when they were in a rush (predicted odds = 0.32; see S1 Table in S1 File).

All seven of the significant predictors (i.e., both those that predicted eating unhealthily and those that predicted eating healthily) were then entered together into an overall model. Meal planning was the only predictor that remained significant in the overall model for eating unhealthily (*b* = -1.13, *SE* = 0.22, *p* < .001), and having no other food options was the only predictor that remained significant for eating healthily (*b* = -0.65, *SE* = 0.27, *p* = .017), indicating that these factors predicted a significant proportion of the variance in healthiness over and above the other predictors.

#### Comparison to non-exercise day random meal sample

The same contextual-factors analyses were then repeated in the random eating occasion sample from non-exercise days (*n* = 593) to examine whether the predictors that were identified for post-exercise meal healthiness were specific to meals following exercise, or whether the same predictors also predicted healthiness of meals that did not follow exercise. Regarding eating unhealthily, none of the contextual factors that predicted unhealthy eating for post-exercise meals (feeling hungry, being in a bad mood, planning to eat the food, food availability, and eating at home) predicted healthiness of non-exercise random meals (*p*s > .050). Similarly, none of the contextual factors that predicted meal healthiness in the post-exercise meal sample predicted meal healthiness in the non-exercise random meals (*p*s > .050). (See S2 Table in S1 File for full results.)

*Characteristics of exercise*. Characteristics of exercise (exercise intensity, exercise duration, exercise type) were tested as predictors of healthiness of post-exercise meals. However, none of the characteristics of exercise were significant predictors of post-exercise meal healthiness for eating unhealthily or for eating healthily (*p*s > .050; see S3 Table in S1 File).

### Discussion

Over the 28-day study period, there was no evidence that participants compensated for exercise by eating less healthily when averaging across participants. Rather, participants were relatively *less* likely to eat unhealthily after exercise compared to random meals drawn from non-exercise days. Participants also ate proportionally fewer unhealthy meals on exercise days compared to non-exercise days. Of note, however, there was also individual variability in patterns of eating behaviour over time.

Although participants in this study did not, on average, compensate by eating less healthily after exercise, they did eat relatively more main meals on exercise days than on non-exercise days (snack intake was not influenced by exercise). This finding suggests that people might be compensating for their exercise by changing the *amount* of food that they eat, rather than by making unhealthy food choices after exercise. If the meals that people eat on exercise days are the same size (or larger) than what they usually eat, and they are also eating more meals overall, then this would result in a net increase in total food consumed on exercise days. It is also possible, however, that people eat smaller meals throughout the day on exercise days, but do not consume more food overall. This issue is explored in Study 2.

A secondary aim of the current study was to explore whether any contextual factors or characteristics of exercise influenced the healthiness of post-exercise meals. For eating unhealthily, only meal planning remained a significant predictor in the overall model, such that such that unplanned meals were relatively more likely to be unhealthy. For eating healthily, having no other food options available remained significant in the overall model. There was no evidence that the characteristics of exercise (intensity, duration, type) predicted the healthiness of post-exercise meals. Further research is needed to substantiate these exploratory findings.

## Study 2

Study 1 focused on the healthiness of the meals consumed, but it is also important to consider the size of the meals consumed. For example, people might compensate by eating a larger amount of food after exercise, rather than by eating less healthily. In line with this idea, Study 1 also found that participants consumed more main meals (but not more snacks) on exercise days compared to non-exercise days. Consistent consumption of larger portions post exercise could hinder weight-loss attempts by offsetting some of the caloric deficit created through exercise [10] and potentially even lead to weight gain [40]. A pattern of consuming larger portions of food after exercise might be particularly detrimental for those who tend to eat unhealthily after exercise, given that poor diet has numerous associated health risks (e.g., [41]).

Previous laboratory studies have shown that participants consumed larger amounts of unhealthy food after exercise when the exercise was perceived as more effortful [13, 14]. However, the foods provided to participants were predominately unhealthy, and it is possible that this unhealthy eating effect might be an artefact of predominately unhealthy food being available. That is, consumption of larger amounts of unhealthy food after exercise in these studies might simply reflect consumption of a greater amount of whatever food was available, and not necessarily a motivated increase in unhealthy eating. Therefore, assessing the portion size of meals consumed in everyday life will provide further insight into whether people are eating larger portions after exercise, and broaden current understanding of patterns of post-exercise eating behaviour in general.

The primary aim of Study 2 was to replicate and extend Study 1 by adding a measure of portion size to test whether participants were compensating by eating a larger amount of food after exercise. As in the previous study, participants were asked to report their food intake and exercise behaviour at the end of each day for 28 days. In line with the findings of the previous study, it was hypothesised that participants would, on average, eat less unhealthily at post-exercise meals compared to random meals, and on exercise days compared to non-exercise days. We did not have an expectation about whether exercise would predict the portion size of meals at the post-exercise eating occasion and on exercise days in general. Consistent with Study 1, a secondary aim was to examine whether any contextual factors (e.g., food availability, feeling hungry) or characteristics of exercise (e.g., type, duration, and intensity) influenced the healthiness and size of post-exercise meals.

### Method

#### Participants

Participants (*N* = 67) were either Australian community members recruited via online advertisements (*n* = 48) or undergraduate students at an Australian university (*n* = 19). Eligibility requirements were the same as Study 1 and, as with Study 1, participants who completed fewer than 18 of the 28 end-of-day surveys (*n* = 12) were excluded from analyses, leaving a final sample of 55 participants (40 women, 15 men). The mean age of the sample was 23.49 years (*SD* = 8.38, range = 18–62) and the mean BMI was 23.00 (*SD =* 1.52; 16.97–36.31). Regarding ethnicity, 67% identified as Asian, 22% identified as White, and 11% identified as “other”. The majority of the sample wanted to lose weight (69%), and 45% were dieting or watching what they ate. From the prescreening eligibility measures, participants reported exercising on average 3.00 times per week (*SD* = 0.84 times) in the previous four weeks and eating less healthily after 49.64% of their exercise sessions (*SD* = 14.27%).

#### Procedure

The study protocol was approved by the university’s Human Research Ethics Advisory Panel and the procedure was identical to Study 1 with the following exceptions:

1. Self-reported healthiness of meals: Rather than asking about how healthy the target meals were compared to what they normally eat (as in Study 1), participants were asked to rate the healthiness of each meal on a 5-point scale (1 = *unhealthy*, 5 = *healthy*).
2. Self-reported portion size: Participants were also asked to rate the size of the meal or snack (1 = *small*, 5 = *big*). A self-reported measure of portion size was used to keep the daily surveys brief. Obtaining validated portion size estimates (e.g., through formal dietary assessment tools) would have been time-consuming to complete and would have been likely to reduce compliance across the 28-day survey period.
3. Student participants were compensated with course credit. Community participants were compensated a maximum of AUD $60 if they completed the initial questionnaire and at least five surveys in each week of the study.

#### Coding of meal healthiness

The meal coding categorisation was the same as described for Study 1. Two coders independently coded all 1,397 surveys (5,585 meals). Cohen’s kappa was .74, indicating moderate agreement [38]. Discrepancies were resolved with a third coder who was blind to the coding completed by the first two coders. A total of 13 meals were excluded because they could not be coded (post-exercise meals = 2; random meals = 11). As in Study 1, meal healthiness as coded from participants’ meal descriptions was significantly correlated with self-reports of meal healthiness, *r* = .69, *p* < .001.

#### Statistical analysis

The main analyses examined whether exercise was a predictor of the *healthiness* of the meals consumed and, separately, whether exercise was a predictor of the *portion size* of the meals consumed. Healthiness was assessed at both the meal level and day level, as in Study 1. Regarding amount of food consumed, compensatory eating was again explored at the meal level such that the subset of post-exercise meals (*n =* 442 meals) was compared to randomly-selected meals from non-exercise days (*n =* 812 meals) to determine whether exercise predicted the portion size of the meal (self-reported by participants, 1 = *small*, 5 = *big*). Compensatory eating was also examined at the day level by testing whether exercise (yes/no) was a predictor of the proportion of meals within a day that were rated as “somewhat big” or “big”. As in Study 1, we tested whether exercise (yes/no) predicted the number of main meals and snacks consumed (i.e., the frequency of eating).

The next set of analyses focused on the post-exercise meal subset to determine whether the healthiness (and, separately, portion size) was predicted by any contextual factors or characteristics of the exercise, in line with Study 1.

## Results

### Descriptive findings

There were a total of 1,397 surveys recorded. Participants completed 25.40 surveys (*SD* = 2.73) on average across the 28-day survey period, and 93% of surveys were completed on time (i.e., the same evening). The total number of eating occasions recorded was 5,585, with participants consuming 2.59 main meals (*SD* = 1.29) and 1.40 snacks (*SD =* 1.29) per day on average.

*Exercise sessions*. There were 573 total days on which participants reported exercising, and participants exercised an average of 10.42 days (*SD* = 5.50 days) during the 28-day survey period. Participants predominately reported engaging in only one exercise session per day (*n =* 469), although there were some days on which participants completed two sessions (*n* = 94) or more than two sessions (*n* = 10). The analyses focused on the first exercise occasion of the day, of which 50.96% of sessions were described as aerobic/cardio, 19.02% were strength, 13.96% were balance or flexibility, 6.81% were sport, and 9.25% were a combination. The modal duration of these exercise sessions was 30–60 min. Regarding exercise intensity, 19.37% of sessions were vigorous, 42.41% were moderate, and 38.22% were low intensity.

*Meals*. There were 444 eating occasions that fell after an exercise session (there were no post-exercise meals on 129 days). Of the post-exercise meals that could be coded for healthiness (*n* = 442), 26.24% were unhealthy, 45.02% were mixed, and 28.73% were healthy. Regarding self-reported portion size, 9.00% of post-exercise meals were small, 12.84% were somewhat small, 48.42% were moderate, 20.95% were somewhat big, and 8.78% were big.

One random meal was captured from each of the non-exercise days (*n* = 823) to compare to the post-exercise meal subset. Of those meals that could be coded for healthiness (*n =* 812), 34.98% were unhealthy, 34.73% were mixed, and 30.29% were healthy. Regarding self-reported portion size, 12.41% of random meals were small, 18.25% were somewhat small, 46.23% were moderate, 15.69% were somewhat big, and 7.42% were big (see S1 Fig in S1 File).

**Fig 2:**
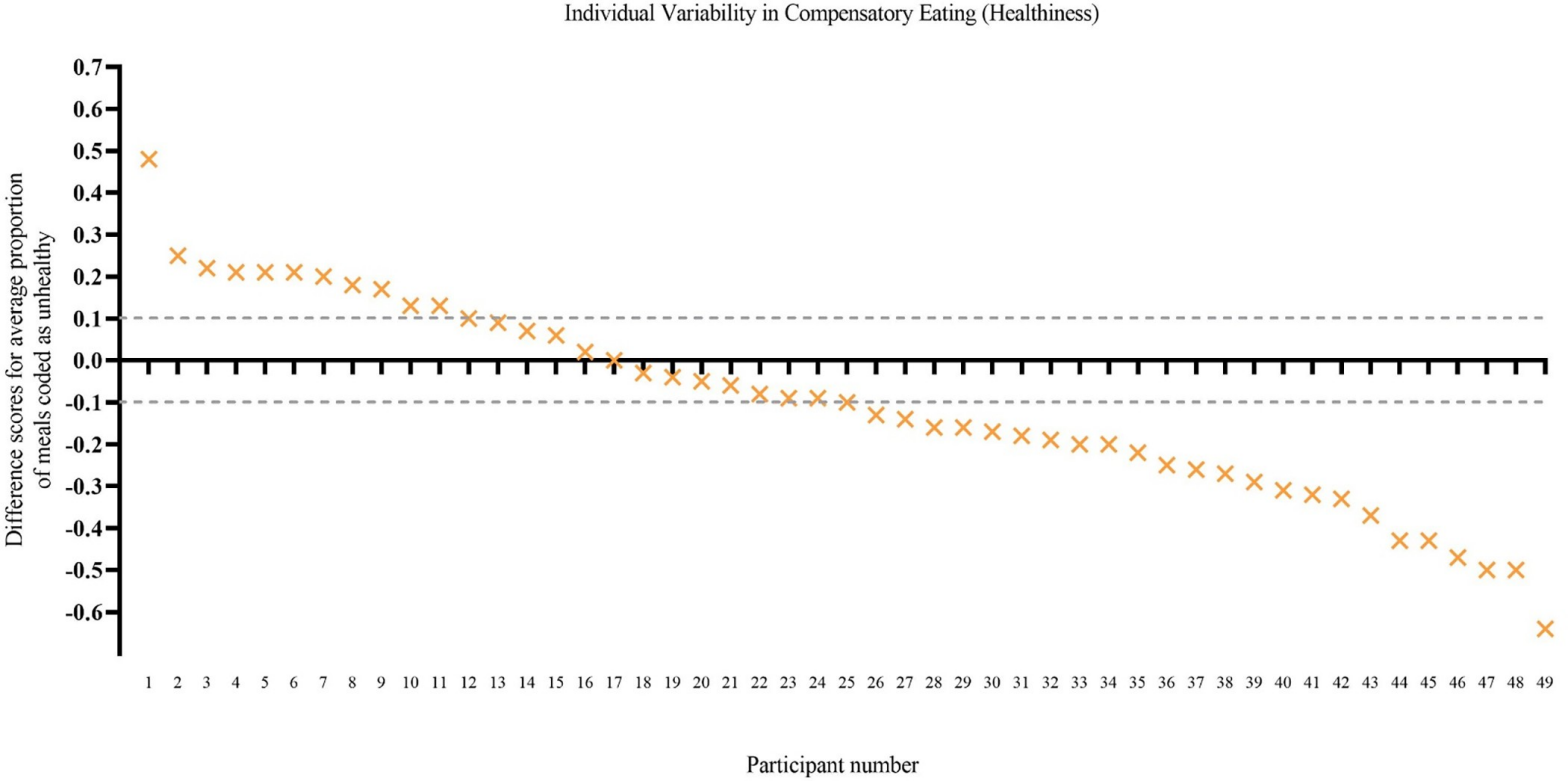
Individual variability in patterns of eating—Healthiness (Study 2). The y axis shows a difference score for each participant: the proportion of post-exercise meals that that were unhealthy minus the proportion of random meals that were unhealthy. A positive score indicates that, on average, the participant consumed a greater proportion of unhealthy meals post exercise than at random meals (i.e., compensatory eating). Dotted lines indicate the boundaries (± 0.10) used to mark an eating pattern that was indifferent to exercise (similar proportion of unhealthy meals after exercise and at random meals). Participant scores are ordered in descending order from most compensatory eating to least compensatory eating. Difference scores could only be calculated for 49 of the 55 participants because six did not have any post-exercise meals.

*Individual variability*. There was considerable individual variability in the pattern of eating behaviours in terms of both the healthiness of meals consumed and the amount of food consumed. For each participant, the proportion of post-exercise meals coded as unhealthy was compared to the proportion of non-exercise day random meals coded as unhealthy. Eleven participants showed a pattern of compensatory eating behaviour (eating proportionally more unhealthy post-exercise meals than random meals) whereas 24 showed the opposite pattern (eating proportionally fewer unhealthy post-exercise meals than random meals), and 14 showed an indifferent eating pattern (similar proportions—no more than 10% difference—in unhealthy post-exercise meals and unhealthy non-exercise day random meals; see Fig 2).

**Fig 3:**
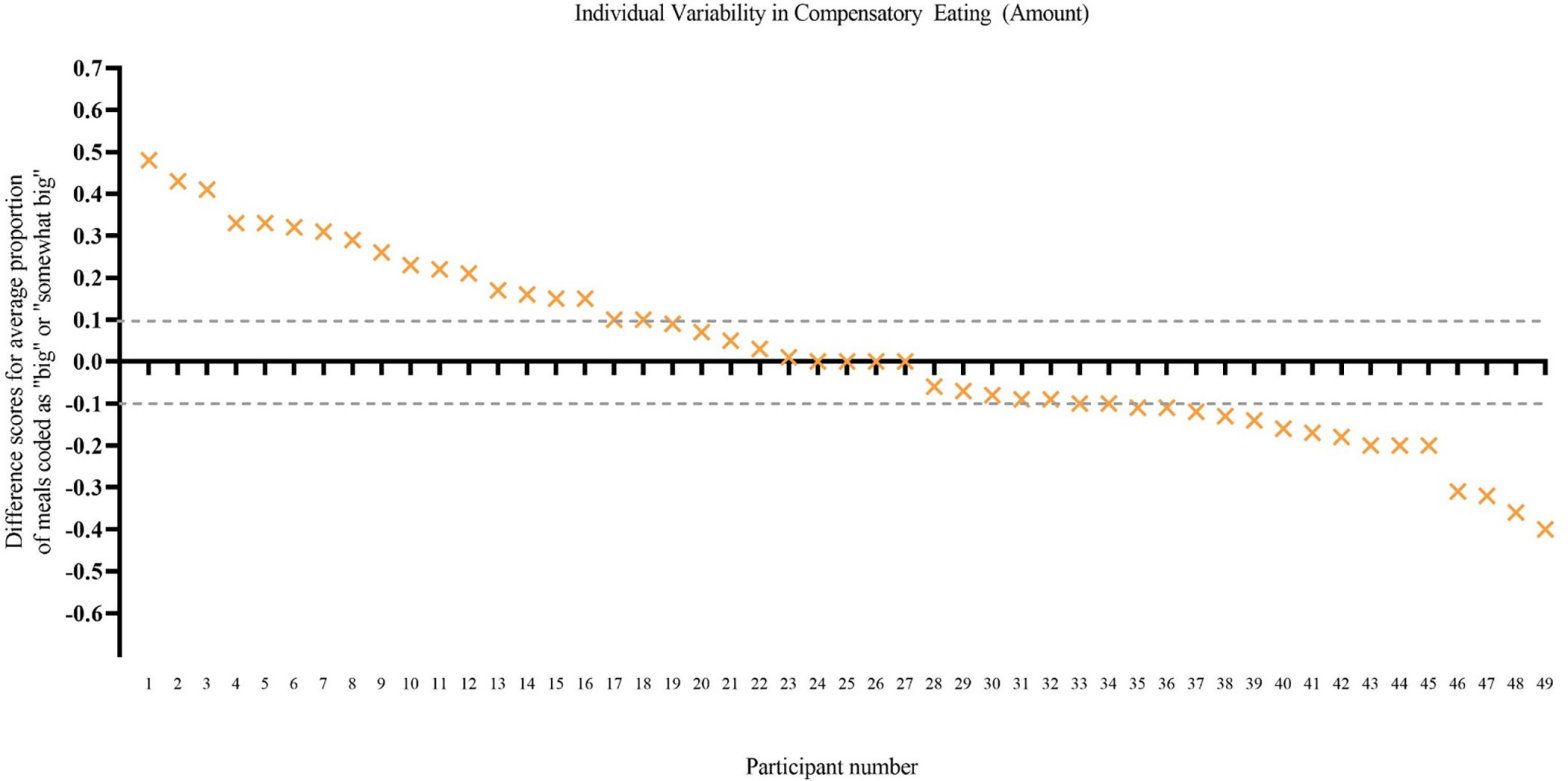
Individual variability in patterns of eating—Amount (Study 2). The y axis shows a difference score for each participant: the proportion of post-exercise meals that were big or somewhat big minus the proportion of random meals that were big or somewhat big. A positive score indicates that, on average, the participant consumed a greater proportion of big meals post exercise than at random meals (i.e., compensatory eating). Dotted lines indicate the boundaries (± 0.10) used to mark an eating pattern that was indifferent to exercise (similar proportion of big meals after exercise and at random meals). Participant scores are ordered in descending order from most compensatory eating to least compensatory eating.

The proportion of post-exercise meals classified as big or somewhat big was also compared to the proportion of non-exercise day random meals classified as big or somewhat big for each participant. Sixteen participants showed a pattern of compensatory eating (eating proportionally more meals classified as big after exercise than at random meals), whereas 15 showed the opposite pattern (eating proportionally fewer meals classified as big after exercise than at random meals), and 18 showed an indifferent eating pattern (i.e., within a 10% difference in the proportion of big meals post exercise than at random meals; see Fig 3).

There was no significant correlation between difference scores for the healthiness of meals and difference scores for the portion size of meals (*r* = .22, *p* = .135). This lack of correlation suggests that the extent to which an individual showed compensatory eating in terms of eating unhealthily after exercise (compared to random meals) was unrelated to the extent to which they consumed larger portions post exercise (compared to at random meals).

#### Exercise and healthiness of meals

At the meal level, exercise was a significant predictor of eating unhealthily such that participants were relatively less likely to eat unhealthily at post-exercise eating occasions (predicted odds = 0.54) compared to random eating occasions on non-exercise days (predicted odds = 0.99). Exercise was also a significant predictor of eating healthily such that participants were relatively less likely to eat healthily at post-exercise eating occasions (predicted odds = 0.60) than at random eating occasions on non-exercise days (predicted odds = 0.85; see Table 4).

**Table 4:** Exercise as a predictor of meal healthiness (Study 2).

		Odds ratio	Lower limit	Upper limit	*b*	*SE*	*t*	*p*
Unhealthy vs. mixed meals								
	Intercept^a^	0.99	0.77	1.26	-0.01	0.12	-0.12	.904
	Post-exercise eating occasion	**0.55**	**0.39**	**0.77**	**-0.60**	**0.17**	**-3.51**	**< .001**
Healthy vs. mixed meals								
	Intercept^a^	0.85	0.68	1.07	-0.16	0.11	-1.40	.168
	Post-exercise eating occasion	**0.71**	**0.54**	**0.92**	**-0.35**	**0.13**	**-2.59**	**.010**

*Note*. Meal healthiness was a multicategorical outcome variable with three categories (unhealthy, mixed, healthy), generating two comparisons against mixed, the reference category: (1) Unhealthy vs. mixed meals and (2) Healthy vs. mixed meals. Exercise was a dichotomous predictor (1 = post-exercise eating occasion, 0 = random eating occasion on non-exercise day). Bold denotes that the predictor was significant.

^a^Intercept of the hierarchical generalized linear model (HGLM) refers to when the value of both the exercise predictor = 0 (i.e., random eating occasion on non-exercise day) and meal healthiness = 0 (i.e., mixed, reference category).

At the day level, exercise was a significant predictor of the percentage of unhealthy eating occasions, such that participants ate proportionally fewer unhealthy meals on exercise days (predicted value = 30.52%) than on non-exercise days (predicted value = 33.90%; see Table 5).

**Table 5:** Exercise as a predictor of the percentage of unhealthy meals in a day (Study 2).

	*b*	*SE*	*t*	*p*
Intercept^a^	33.90	2.05	16.54	< .001
Exercise	**-3.39**	**1.69**	**-2.00**	**.046**

*Note*. Percentage of eating occasions that day coded as unhealthy was a continuous outcome (0%–100%). Exercise was a dichotomous predictor (1 = yes, 0 = no). Bold denotes that the predictor was significant.

^a^Intercept of the hierarchical linear model (HLM) refers to when the exercise = 0 (non-exercise day).

#### Exercise and amount of food consumed

*Portion size of meals*. At the meal level, exercise was a significant predictor of portion size, such that participants reported consuming larger meals post exercise (predicted value = 3.10) than at random meals on non-exercise days (predicted value = 2.86; see Table 6).

**Table 6:** Exercise as a predictor of self-reported portion size of meals (Study 2).

	*b*	*SE*	*t*	*p*
Intercept^a^	2.86	0.06	44.74	< .001
Post-exercise eating occasion	**0.25**	**0.07**	**3.46**	**< .001**

*Note*. Self-reported portion size was treated as a continuous outcome (1 = *small*, 5 = *big*). Exercise was a dichotomous predictor (1 = post-exercise eating occasion, 0 = random eating occasion on non-exercise day). Bold denotes that the predictor was significant.

^a^Intercept of the hierarchical linear model (HLM) refers to when the exercise = 0 (random eating occasion on non-exercise day).

At the day level, exercise was a borderline significant predictor of portion size (*p* = .050). However, the pattern contrasted with the meal-level findings: A relatively smaller proportion of meals were classified as big or somewhat big on exercise days (predicted value = 23.47%) than on non-exercise days (predicted value = 25.97%; see Table 7).

**Table 7:** Exercise as a predictor of the percentage of meals within the day self-rated as big/somewhat big (Study 2).

	*b*	*SE*	*t*	*p*
Intercept^a^	25.97	2.46	10.53	< .001
Exercise	-2.49	1.27	-1.96	.050

*Note*. Percentage of eating occasions that day coded as big/somewhat big was a continuous outcome (0%–100%). Exercise was a dichotomous predictor (1 = yes, 0 = no).

^a^Intercept of the hierarchical linear model (HLM) refers to when exercise = 0 (non-exercise day).

*Frequency of meals*. Exercise was a significant predictor of the number of main meals consumed such that participants ate relatively more main meals on exercise days (predicted event rate = 2.66 main meals) than on non-exercise days (predicted event rate = 2.55 main meals). However, exercise was not a significant predictor of the number of snacks consumed (see Table 8).

**Table 8:** Exercise as a predictor of number of main meals and snacks consumed (Study 2).

		Event rate ratio	Lower limit	Upper limit	*b*	*SE*	*t*	*p*
Main meals								
	Intercept^a^	2.55	2.44	2.68	0.94	0.02	39.61	< .001
	Exercise	**1.04**	**1.01**	**1.07**	**0.04**	**0.02**	**2.46**	**.014**
Snacks								
	Intercept^a^	1.38	1.15	1.64	0.32	0.09	3.63	< .001
	Exercise	1.07	0.99	1.16	0.07	0.04	1.81	.071

*Note*. Main meals was a count variable of the number of main meals (breakfast, lunch, dinner) eaten in one day. Snacks was a count variable of the number of snacks eaten in one day. Exercise was a dichotomous predictor (1 = yes, 0 = no). Bold denotes that the predictor was significant.

^a^Intercept of the HGLM refers to when the value of the exercise predictor = 0 (non-exercise days).

#### Predictors of post-exercise meal healthiness

Contextual factors and characteristics of exercise were examined as predictors of meal healthiness in the post-exercise meal subset.

*Contextual predictors*. For the post-exercise eating occasion subset, there were two significant individual predictors of eating unhealthily. Participants were more likely to eat unhealthily when the meal was not planned (predicted odds = 0.77) compared to when the meal was planned (predicted odds = 0.39). They were also more likely to eat unhealthily when they had cravings for the food (predicted odds = 0.97) compared to when they did not have cravings (predicted odds = 0.38). None of the predictors were significant for eating healthily (see S4 Table in S1 File).

Both individual predictors remained significant when entered together into an overall model, indicating that they each predicted a significant proportion of the variance in healthiness over and above that explained by the other predictor. For meal planning, *b* = -0.72, *SE* = 0.31, *p* = .020, and for cravings, *b* = 0.97, *SE* = 0.28, *p* < .001.

#### Comparison to non-exercise day random meal sample

The contextual factors analyses were repeated for the random eating occasion sample from non-exercise days (*n* = 812). As in the post-exercise sample, unplanned meals were more likely to be unhealthy (predicted odds = 1.44) than were planned meals (predicted odds = 0.55), and participants were more likely to eat unhealthily when they had cravings for the food (predicted odds = 1.60) rather than no cravings (predicted odds = 0.73; see S5 Table in S1 File).

*Characteristics of exercise*. Characteristics of exercise (exercise intensity, exercise duration, exercise type) were tested as predictors of meal healthiness of post-exercise meals. Neither exercise duration nor intensity were significant predictors of post-exercise meal healthiness for eating unhealthily. However, for exercise type, one of the dummy-coded predictors was significant for eating unhealthily. Combination exercise was relatively more likely to be followed by an unhealthy meal (predicted odds = 0.89) than was balance/flexibility exercise (predicted odds = 0.15). However, none of the other types of exercise (cardio, strength, or sport) significantly predicted post-exercise meal healthiness (in comparison to combination exercise). Regarding eating healthily, none of the characteristics of exercise were significant predictors of eating healthily compared to mixed (see S6 Table in S1 File).

#### Predictors of post-exercise meal portion size

Predictors of post-exercise meal portion size were examined using the self-reported measure of portion size (1 = *small*, 5 = *big*), which was treated as a continuous outcome variable.

*Contextual predictors*. The 11 contextual factors referring to parameters of the meal were tested as predictors of post-exercise portion size. Hunger was a significant predictor such that participants ate relatively larger portions after exercise when they were hungry (predicted value = 3.18) compared to when they were not hungry (predicted value = 2.66). They also ate larger portions when eating with other people (predicted value = 3.27) compared to when eating alone (predicted value = 2.83) and when they had to go out of their way to obtain the food (predicted value = 3.39) compared to when it was readily available (predicted value = 2.98; see S7 Table in S1 File).

When all three of these individually significant predictors were added into an overall model, hunger (*b* = 0.48, *SE* = 0.11, *p* < .001), eating alone (*b* = -0.37, *SE* = 0.09, *p* < .001), and food availability (*b* = -0.31, *SE* = 0.12, *p* = .007) all remained significant.

#### Comparison to non-exercise day random meal sample

As with the post-exercise meal sample, hunger, eating alone, and food availability were all significant predictors of portion size in the non-exercise random meal sample. Participants ate relatively larger portions when hungry (predicted value = 2.98) compared to not hungry (predicted value = 2.63), when eating with others (predicted value = 2.99) compared to eating alone (predicted value = 2.71), and when they had to go out of their way to obtain the food (predicted value = 3.21) compared to when it was readily available (predicted value = 2.73; see S8 Table in S1 File).

*Characteristics of exercise*. Characteristics of exercise (intensity, type, duration) were tested as predictors of post-exercise portion size. None of the predictors were significant (*p*s > .050; see S9 Table in S1 File).

### Discussion

As in Study 1, participants were less likely to eat an unhealthy meal after exercise than they were to eat an unhealthy meal at random meals on non-exercise days, and participants also ate proportionally fewer unhealthy meals on exercise days than on non-exercise days. In addition, participants were also less likely to eat a *healthy* meal compared to a mixed meal, which might suggest that participants ate less “extremely” after exercise or reflect between-participant variability in eating patterns.

Study 2 also examined the amount of food eaten. We found that participants consumed larger meals post-exercise than at random meals, in contrast with evidence from a meta-analysis which found that energy intake did not significantly increase after exercise [10]. However, this meta-analysis included only laboratory studies, unlike our naturalistic method of assessment. We also found that participants consumed a *smaller* proportion of big meals on exercise days than on non-exercise days. That is, there was compensatory eating with regard to consumption of larger portions after exercise at the meal level, but not at the day level, which might suggest that people may be making up for larger post-exercise meals by eating smaller meals the rest of the day. Note, however, that the day-level effect was borderline significant, so these results must be interpreted with caution. Adjusting for large post-exercise meals by eating less at other meals also seems unlikely because studies examining portion size have consistently found that, when people overeat due to the experimentally-manipulated presence of large portion sizes, they do not tend to modify their portion sizes to be smaller at subsequent meals [4, 40, 42].

Regarding contextual predictors, post-exercise meals and random meals were both more likely to be unhealthy when the meal was not planned, and when cravings were present. Similarly, regarding predictors of meal size, both post-exercise and random meals were likely to be larger when participants reported that they were hungry, eating with others, and when they had to go out of their way to obtain the food.

## General discussion

Previous research on compensatory eating has not examined consistency in post-exercise eating patterns over time, despite preliminary evidence to suggest variability in compensatory eating both between individuals (e.g., [15]), and within the same individual across multiple exercise occasions (e.g., [20]). The present studies fill this gap by investigating compensatory eating behaviour after exercise over 28 days in a naturalistic setting using daily diary methods. Across both studies, there was no indication that participants consistently compensated for exercise by eating less healthily afterwards. Instead, participants ate more healthily on average at post-exercise meals compared to random meals, and ate proportionally more healthy meals on exercise days compared to non-exercise days. These findings are at odds with lab studies that demonstrate evidence of compensatory eating after influencing perceptions of exercise [11, 13, 14]. However, the findings are consistent with other research showing no difference in intake after exercise compared to after rest [15], or no difference in intake after exercise compared to no-exercise [12, 43]. Notably, there was also substantial variability across participants in the healthiness of post-exercise meals. In both studies, approximately 25% of participants tended to compensate by eating more unhealthily on exercise days than they did on non-exercise days, while others showed the opposite pattern, and a third group’s eating behaviour appeared not to be affected by exercise. These results build on previous research which has demonstrated that there is variability in people’s eating behaviour after a single exercise occasion [16, 17], and suggests that there may be meaningful differences in people’s patterns of exercise-associated eating behaviour when examining behaviour over longer time periods.

The results of Study 2 also showed that participants consumed relatively bigger portions at post-exercise meals than they did at random meals on non-exercise days over a 28-day period. Although inconsistent with evidence from a meta-analysis of laboratory studies [10], this finding is consistent with the only previous study to investigate post-exercise eating behaviour in a naturalistic setting, which found that people consumed more food in the three hours after exercise than in an equivalent period of time on non-exercise days [27]. When considered in combination with the findings pertaining to the healthiness of post-exercise meals, these results suggest that, on average, people might be consuming larger portions after exercise, but that the type of food consumed post exercise might be more healthy than meals on non-exercise days. Thus, even if people are eating larger portions after exercise, this may not be of great concern because they may be consuming relatively nutritious foods. However, there does appear to be variability in the healthiness of meals consumed post exercise, and consuming larger portions could be more problematic for some people if those portions frequently consist of unhealthy foods. Consumption of unhealthy food also has negative health implications, regardless of weight [41].

Both studies also found that participants ate more main meals on exercise days, but that exercise was not a significant predictor of number of snacks consumed. This finding may reflect variation in how people define meals and snacks, which may depend on the timing or patterning of eating occasions, the nutritional composition of eating occasions, and the context of eating occasions [44, 45]. For example, if healthy foods are relatively more likely to be classified as a main meal than a snack, then the consumption of more main meals on exercise days might reflect a change in classification of meals versus snacks, rather than a true increase in total number of eating occasions. Similarly, if larger portions of the same kind of food are more likely to be classified as main meals than snacks, the finding that more main meals were consumed on exercise days might reflect the consumption of larger portions after exercise.

A secondary aim of both studies was to examine whether any contextual factors or characteristics of exercise itself influenced the healthiness (Study 1 and 2) and portion size (Study 2) of post-exercise meals, but there was no consistent pattern to the results across studies. In Study 1, lack of meal planning was a unique predictor of eating unhealthily that was specific to post-exercise meals. Although lack of meal planning and having cravings were significant predictors in Study 2, they were not unique to post-exercise meals (i.e., they predicted the healthiness of random meals as well). For the portion size of post-exercise meals, larger meals were more likely when participants were feeling hungry, eating with others, and when food for that meal was not readily available. Again, these predictors were not specific to post-exercise meals because they also significantly predicted portion size for random meals on non-exercise days. In both studies, none of the characteristics of exercise (intensity, duration, type) predicted the healthiness or the portion size of post-exercise meals, with the exception that one of the dummy-coded predictors for exercise type was a significant predictor of coded meal healthiness in Study 2.

### Limitations and future directions

The current work built on previous daily life method studies assessing contingencies between health behaviours [30, 31] by exploring post-exercise eating behaviour over an extended time period. In both our studies, the samples consisted predominately of women, many of whom reported wanting to lose weight and were currently dieting or watching what they ate. We also limited our study to people who were moderately frequent exercisers and self-reported eating unhealthily after exercise at least some of the time so that we could have enough instances of the behaviours of interest to be able to address our research questions. However, by only including participants who exercised at least moderately frequently, we may not have captured the patterns of post-exercise eating of people who exercise only occasionally or who exercise almost every day. By only including participants who self-reported eating unhealthily at least some of the time, our results may actually overestimate the occurrence of post-exercise unhealthy eating. Further research is needed to test the generalisability of these findings in more diverse samples.

Daily diaries have the advantage of providing an ecologically valid way of exploring behaviour. However, one limitation associated with these methods is that simply asking participants to record their food intake and exercise behaviour could lead to changes in their behaviour compared to when their behaviour is not monitored [46]. There is currently no viable non-invasive alternative to asking participants to self-monitor food intake, but future studies could potentially use wearable activity trackers to monitor exercise (for a review, see [47]). Another limitation related to the diary method used is that, in order to increase compliance and maintain engagement over the 28-day study period, we opted for a brief report of food intake and exercise, rather than obtaining detailed assessments (with its associated participant burden). Despite strong concordance between the coded healthiness of the meals and participants’ self-reports of those meals, future research could assess dietary intake, portion size, and physical activity using more detailed and precise assessments (such as computer-based food recognition technology, once it becomes more refined and feasible; [48, 49]). More precise measures of food intake and physical activity could provide further detail about the nature of mixed meals and how meal composition may be influenced by exercise or exercise type. These more detailed assessment approaches could also provide information about the interplay between the size and the healthiness of meals consumed post-exercise, including whether exercise influences overall energy intake in addition to the size or healthiness of the meal alone.

A limitation associated with our analytical approach was the choice to use random meals on non-exercise days as our comparison, given that participants’ exercise sessions (and subsequent meals after exercising) were likely not occurring at random times. It is possible that the timing of exercise in the day might have influenced the size or healthiness of post-exercise meals. If, for example, for some participants exercise systematically occurred prior to a main meal (which may be larger than a snack) or prior to dessert (which may be less healthy than other meals), then random meals drawn from any time throughout the day are not an optimal comparison. However, given that there was some variation in the timing of exercise both within and between participants, our analytic approach is unlikely to have had a systematic impact on the overall results, but rather to have introduced noise into the data. In order to address this limitation, future research could examine compensatory eating in daily life using artificial intelligence or adaptive modelling approaches to identify “yoked” meals on non-exercise days that most closely matched the timing of post-exercise meals for each participant, rather than comparing post-exercise meals to random meals.

These studies also raise important questions for future research around the time course of compensatory eating. It is possible that compensatory eating might occur at other times, rather than at the meal immediately following exercise. That is, people might compensate by eating unhealthily or eating more food at a later meal that day, or even at a meal the next day. Although eating behaviour on exercise days was compared to eating behaviour on non-exercise days, this measure might not have been precise enough to capture any temporal links between exercise and compensatory eating that occurred at a later time. It is also possible that people may engage in compensatory eating when they are expecting to exercise later in the day (a sort of pre-compensation). Indeed, one study has shown that some people increase their food intake when they are anticipating future exercise [50]. Future research should therefore explore the time course of compensatory eating with greater precision.

## Conclusion

The current studies used daily diary methods to explore compensatory eating after exercise in everyday life over an extended time period. Using this approach, we found that participants were less likely to eat unhealthily after exercise compared to at random meals on non-exercise days, and a smaller proportion of meals were unhealthy on exercise days than on non-exercise days. Study 2 also found that participants consumed larger portions of food at post-exercise meals compared to random meals on non-exercise days. Considered together, these findings suggest that, on average, people might eat larger portions of healthier food after exercise in their everyday life. There was, however, considerable individual variability in patterns of eating behaviour post exercise. Future research should corroborate these findings in more diverse samples and with more precise dietary assessment methods in order to understand the composition of post-exercise meals, and the factors that influence between- and within-individual differences in exercise-related eating habits. Broadening current understanding of compensatory eating after exercise has the potential to facilitate development of strategies to improve health behaviour regulation.

## Supporting information

S1 File (DOCX)
