# Supplementary material for: Compensatory eating after exercise in everyday life: Insights from daily diary studies
Source: PLoS One. 2023 Mar 15;18(3):e0282501. doi: 10.1371/journal.pone.0282501 (PMC10016725; doi:10.1371/journal.pone.0282501)
Supplement: S1 File — (DOCX) [file pone.0282501.s001.docx]

S1 Table: Contextual Predictors of Post-Exercise Meal Healthiness (Study 1)

|  | | Odds ratio | Lower limit | Upper limit | *b* | *SE* | *t* | *p* |
| --- | --- | --- | --- | --- | --- | --- | --- | --- |
| Unhealthy vs. mixed | |  |  |  |  |  |  |  |
|  | Intercept^a^ | 1.04 | 0.52 | 2.09 | 0.04 | 0.35 | 0.12 | .907 |
|  | Feeling hungry | **0.45**^b^ | **0.22** | **0.93** | **-0.79** | **0.36** | **-2.17** | **.031** |
| Healthy vs. mixed | |  |  |  |  |  |  |  |
|  | Intercept^a^ | 0.70 | 0.35 | 1.40 | -0.36 | 0.34 | -1.04 | .305 |
|  | Feeling hungry | 0.94 | 0.47 | 1.85 | -0.07 | 0.35 | -0.19 | .848 |
| Unhealthy vs. mixed | |  |  |  |  |  |  |  |
|  | Intercept | 0.52 | 0.36 | 0.75 | -0.65 | 0.18 | -3.56 | < .001 |
|  | Feeling stressed | 2.21 | 0.99 | 4.88 | 0.79 | 0.40 | 1.96 | .051 |
| Healthy vs. mixed | |  |  |  |  |  |  |  |
|  | Intercept | 0.65 | 0.44 | 0.94 | -0.44 | 0.19 | -2.34 | .024 |
|  | Feeling stressed | 1.29 | 0.51 | 3.28 | 0.25 | 0.47 | 0.53 | .594 |
| Unhealthy vs. mixed | |  |  |  |  |  |  |  |
|  | Intercept | 0.52 | 0.37 | 0.75 | -0.65 | 0.18 | -3.59 | < .001 |
|  | In a bad mood | **4.48** | **1.03** | **19.42** | **1.50** | **0.75** | **2.01** | **.045** |
| Healthy vs. mixed | |  |  |  |  |  |  |  |
|  | Intercept | 0.64 | 0.44 | 0.94 | -0.44 | 0.19 | -2.31 | .025 |
|  | In a bad mood | 2.27 | 0.49 | 10.58 | 0.81 | 0.78 | 1.05 | .294 |
| Unhealthy vs. mixed | |  |  |  |  |  |  |  |
|  | Intercept | 0.53 | 0.37 | 0.75 | -0.64 | 0.18 | -3.64 | < .001 |
|  | Feeling tired | 1.23 | 0.70 | 2.18 | 0.21 | 0.29 | 0.72 | .472 |
| Healthy vs. mixed | |  |  |  |  |  |  |  |
|  | Intercept | 0.73 | 0.48 | 1.10 | -0.32 | 0.21 | -1.54 | .131 |
|  | Feeling tired | 0.75 | 0.41 | 1.35 | -0.29 | 0.30 | -0.97 | .331 |
| Unhealthy vs. mixed | |  |  |  |  |  |  |  |
|  | Intercept | 0.62 | 0.43 | 0.90 | -0.47 | 0.18 | -2.61 | .012 |
|  | No other food options | 0.65 | 0.36 | 1.16 | -0.43 | 0.29 | -1.47 | .142 |
| Healthy vs. mixed | |  |  |  |  |  |  |  |
|  | Intercept | 0.74 | 0.49 | 1.12 | -0.30 | 0.20 | -1.48 | .147 |
|  | No other food options | **0.51** | **0.31** | **0.86** | **-0.67** | **0.26** | **-2.57** | **.011** |
| Unhealthy vs. mixed | |  |  |  |  |  |  |  |
|  | Intercept | 1.10 | 0.73 | 1.65 | 0.09 | 0.20 | 0.46 | .648 |
|  | Planned to eat that food | **0.26** | **0.17** | **0.42** | **-1.34** | **0.24** | **-5.61** | **< .001** |
| Healthy vs. mixed | |  |  |  |  |  |  |  |
|  | Intercept | 0.58 | 0.35 | 0.97 | -0.55 | 0.25 | -2.15 | .037 |
|  | Planned to eat that food | 1.19 | 0.62 | 2.26 | 0.17 | 0.33 | 0.52 | .601 |
| Unhealthy vs. mixed | |  |  |  |  |  |  |  |
|  | Intercept | 0.98 | 0.60 | 1.60 | -0.02 | 0.25 | -0.09 | .927 |
|  | Food readily available | **0.50** | **0.29** | **0.87** | **-0.69** | **0.28** | **-2.44** | **.015** |
| Healthy vs. mixed | |  |  |  |  |  |  |  |
|  | Intercept | 0.59 | 0.29 | 1.22 | -0.53 | 0.36 | -1.46 | .152 |
|  | Food readily available | 1.14 | 0.53 | 2.47 | 0.13 | 0.39 | 0.33 | .740 |
| Unhealthy vs. mixed | |  |  |  |  |  |  |  |
|  | Intercept | 0.46 | 0.29 | 0.72 | -0.78 | 0.23 | -3.43 | .001 |
|  | Cravings for food | 1.65 | 0.90 | 3.03 | 0.50 | 0.31 | 1.63 | .103 |
| Healthy vs. mixed | |  |  |  |  |  |  |  |
|  | Intercept | 0.65 | 0.43 | 0.99 | -0.43 | 0.21 | -2.05 | .046 |
|  | Cravings for food | 1.03 | 0.66 | 1.60 | 0.03 | 0.22 | 0.13 | .896 |
|  |  |  |  |  |  |  |  |  |
|  |  |  |  |  |  |  |  |  |
| Unhealthy vs. mixed | |  |  |  |  |  |  |  |
|  | Intercept | 0.71 | 0.49 | 1.03 | -0.35 | 0.19 | -1.86 | .069 |
|  | Eating alone | 0.62 | 0.35 | 1.08 | -0.48 | 0.29 | -1.69 | .092 |
| Healthy vs. mixed | |  |  |  |  |  |  |  |
|  | Intercept | 0.53 | 0.33 | 0.84 | -0.64 | 0.23 | -2.77 | .008 |
|  | Eating alone | 1.50 | 0.87 | 2.59 | 0.40 | 0.28 | 1.46 | .145 |
| Unhealthy vs. mixed | |  |  |  |  |  |  |  |
|  | Intercept | 0.59 | 0.40 | 0.88 | -0.53 | 0.20 | -2.67 | .010 |
|  | In a rush | 0.84 | 0.38 | 1.84 | -0.18 | 0.40 | -0.45 | .656 |
| Healthy vs. mixed | |  |  |  |  |  |  |  |
|  | Intercept | 0.75 | 0.52 | 1.08 | -0.29 | 0.18 | -1.60 | .117 |
|  | In a rush | **0.43** | **0.19** | **0.97** | **-0.85** | **0.42** | **-2.03** | **.043** |
| Unhealthy vs. mixed | |  |  |  |  |  |  |  |
|  | Intercept | 0.87 | 0.56 | 1.34 | -0.14 | 0.22 | -0.66 | .512 |
|  | Eating at home | **0.45** | **0.25** | **0.79** | **-0.80** | **0.29** | **-2.76** | **.006** |
| Healthy vs. mixed | |  |  |  |  |  |  |  |
|  | Intercept | 0.59 | 0.37 | 0.97 | -0.52 | 0.24 | -2.14 | .038 |
|  | Eating at home | 1.14 | 0.64 | 2.03 | 0.13 | 0.29 | 0.44 | .657 |
| *Note.* Meal healthiness was a multicategorical outcome variable with three categories (unhealthy, mixed, healthy), generating two comparisons against mixed, the reference category: (1) Unhealthy vs. mixed meals and (2) Healthy vs. mixed meals. Each of the 11 contextual factors are dichotomous predictors (1 = yes, 0 = no). They were examined as individual predictors. Bold denotes that the predictor was significant.  ^a^Intercept of the hierarchical generalized linear model (HGLM) refers to when the value of the contextual predictor (i.e., feeling hungry) = 0 (i.e., not hungry). | | | | | | | | |

S2 Table: Contextual Predictors of Non-exercise Day Meal Healthiness (Study 1)

|  | | Odds ratio | Lower limit | Upper limit | *b* | *SE* | *t* | *p* |
| --- | --- | --- | --- | --- | --- | --- | --- | --- |
| Unhealthy vs. mixed | |  |  |  |  |  |  |  |
|  | Intercept^a^ | 1.11 | 0.77 | 1.60 | 0.10 | 0.18 | 0.57 | .574 |
|  | Feeling hungry | 0.85 | 0.53 | 1.34 | -0.16 | 0.24 | -0.66 | .510 |
| Healthy vs. mixed | |  |  |  |  |  |  |  |
|  | Intercept^a^ | 0.57 | 0.38 | 0.86 | -0.57 | 0.21 | -2.76 | .008 |
|  | Feeling hungry | 1.28 | 0.77 | 2.14 | 0.25 | 0.26 | 0.96 | .338 |
| Unhealthy vs. mixed | |  |  |  |  |  |  |  |
|  | Intercept | 0.76 | 0.55 | 1.05 | -0.28 | 0.16 | -1.71 | .094 |
|  | Feeling stressed | **3.24^b^** | **2.01** | **5.21** | **1.17** | **0.24** | **4.85** | **< .001** |
| Healthy vs. mixed | |  |  |  |  |  |  |  |
|  | Intercept | 0.69 | 0.52 | 0.92 | -0.37 | 0.14 | -2.57 | .014 |
|  | Feeling stressed | 0.93 | 0.55 | 1.56 | -0.07 | 0.27 | -0.28 | .782 |
| Unhealthy vs. mixed | |  |  |  |  |  |  |  |
|  | Intercept | 0.93 | 0.71 | 1.22 | -0.07 | 0.14 | -0.55 | .585 |
|  | In a bad mood | 1.80 | 0.87 | 3.73 | 0.59 | 0.37 | 1.58 | .114 |
| Healthy vs. mixed | |  |  |  |  |  |  |  |
|  | Intercept | 0.87 | 0.48 | 1.56 | -0.14 | 0.30 | -0.48 | .633 |
|  | In a bad mood | 0.69 | 0.51 | 0.93 | -0.37 | 0.15 | -2.54 | .014 |
| Unhealthy vs. mixed | |  |  |  |  |  |  |  |
|  | Intercept | 0.79 | 0.57 | 1.09 | -0.24 | 0.16 | -1.46 | .151 |
|  | Feeling tired | **1.75** | **1.14** | **2.69** | **0.56** | **0.22** | **2.57** | **.010** |
| Healthy vs. mixed | |  |  |  |  |  |  |  |
|  | Intercept | 0.75 | 0.54 | 1.05 | -028 | 0.16 | -1.73 | .091 |
|  | Feeling tired | 0.71 | 0.42 | 1.22 | -0.34 | 0.27 | -1.23 | .219 |
| Unhealthy vs. mixed | |  |  |  |  |  |  |  |
|  | Intercept | 0.94 | 0.68 | 1.30 | -0.06 | 0.16 | -0.39 | .696 |
|  | No other food options | 1.21 | 0.79 | 1.87 | 0.19 | 0.22 | 0.88 | .379 |
| Healthy vs. mixed | |  |  |  |  |  |  |  |
|  | Intercept | 0.71 | 0.53 | 0.95 | -0.35 | 0.14 | -2.41 | .020 |
|  | No other food options | 0.87 | 0.54 | 1.41 | -0.14 | 0.25 | -0.58 | .564 |
| Unhealthy vs. mixed | |  |  |  |  |  |  |  |
|  | Intercept | 1.22 | 0.88 | 1.68 | 0.20 | 0.60 | 1.23 | .225 |
|  | Planned to eat that food | 0.66 | 0.44 | 1.01 | -0.41 | 0.21 | -1.92 | .055 |
| Healthy vs. mixed | |  |  |  |  |  |  |  |
|  | Intercept | 0.41 | 0.28 | 0.61 | -0.88 | 0.19 | -4.65 | < .001 |
|  | Planned to eat that food | **2.22** | **1.34** | **3.69** | **0.80** | **0.26** | **3.11** | **.002** |
| Unhealthy vs. mixed | |  |  |  |  |  |  |  |
|  | Intercept | 0.68 | 0.39 | 1.19 | -0.38 | 0.28 | 1.38 | .175 |
|  | Food readily available | 1.62 | 0.93 | 2.82 | 0.48 | 0.28 | 1.71 | .089 |
| Healthy vs. mixed | |  |  |  |  |  |  |  |
|  | Intercept | 0.68 | 0.44 | 1.05 | -0.39 | 0.22 | -1.78 | .081 |
|  | Food readily available | 1.00 | 0.59 | 1.70 | < 0.01 | 0.27 | 0.01 | .995 |
| Unhealthy vs. mixed | |  |  |  |  |  |  |  |
|  | Intercept | 0.74 | 0.51 | 1.08 | -0.30 | 0.19 | -1.61 | .114 |
|  | Cravings for food | **1.97** | **1.20** | **3.23** | **0.68** | **0.25** | **2.68** | **.008** |
| Healthy vs. mixed | |  |  |  |  |  |  |  |
|  | Intercept | 0.76 | 0.54 | 1.08 | -0.27 | 0.17 | -1.57 | .122 |
|  | Cravings for food | 0.68 | 0.42 | 1.12 | -0.38 | 0.25 | -1.55 | .123 |
| Unhealthy vs. mixed | |  |  |  |  |  |  |  |
|  | Intercept | 1.02 | 0.74 | 1.39 | 0.02 | 0.15 | 0.98 | .922 |
|  | Eating alone | 0.95 | 0.63 | 1.44 | -0.05 | 0.21 | -0.22 | .824 |
| Healthy vs. mixed | |  |  |  |  |  |  |  |
|  | Intercept | 0.75 | 0.51 | 1.10 | -0.29 | 0.19 | -1.53 | .133 |
|  | Eating alone | 0.81 | 0.47 | 1.41 | -0.21 | 0.28 | -0.74 | .460 |
| Unhealthy vs. mixed | |  |  |  |  |  |  |  |
|  | Intercept | 0.98 | 0.73 | 1.33 | -0.02 | 0.15 | -0.12 | .907 |
|  | In a rush | 1.05 | 0.63 | 1.75 | 0.05 | 0.26 | 0.17 | .863 |
| Healthy vs. mixed | |  |  |  |  |  |  |  |
|  | Intercept | 0.76 | 0.57 | 1.03 | -0.27 | 0.15 | -1.80 | .079 |
|  | In a rush | 0.58 | 0.29 | 1.13 | -0.55 | 0.34 | -1.61 | .107 |
| Unhealthy vs. mixed | |  |  |  |  |  |  |  |
|  | Intercept | 1.17 | 0.83 | 1.66 | 0.16 | 0.17 | 0.93 | .356 |
|  | Eating at home | 0.73 | 0.47 | 1.14 | -0.31 | 0.22 | -1.39 | .165 |
| Healthy vs. mixed | |  |  |  |  |  |  |  |
|  | Intercept | 0.68 | 0.44 | 1.05 | -0.39 | 0.22 | -1.79 | .080 |
|  | Eating at home | 1.00 | 0.65 | 1.54 | < 0.01 | 0.22 | 0.01 | .990 |
| *Note.* Meal healthiness was a multicategorical outcome variable with three categories (unhealthy, mixed, healthy), generating two comparisons against mixed, the reference category: (1) Unhealthy vs. mixed meals and (2) Healthy vs. mixed meals. Each of the 11 contextual factors are dichotomous predictors (1 = yes, 0 = no). They were examined as individual predictors. Bold denotes that the predictor was significant.  ^a^Intercept of the hierarchical generalized linear model (HGLM) refers to when the value of the contextual predictor (i.e., feeling hungry) = 0 (i.e., not hungry). | | | | | | | | |

S3 Table: Characteristics of Exercise as Predictors of Post-Exercise Meal Healthiness (Study 1)

|  | | Odds ratio | Lower limit | Upper limit | *b* | *SE* | *t* | *p* |
| --- | --- | --- | --- | --- | --- | --- | --- | --- |
| Unhealthy vs. mixed | |  |  |  |  |  |  |  |
|  | Intercept | 0.56 | 0.39 | 0.82 | -0.57 | 0.18 | -3.12 | .003 |
|  | Intensity | 1.29 | 0.87 | 1.92 | 0.25 | 0.20 | 1.27 | .207 |
| Healthy vs. mixed | |  |  |  |  |  |  |  |
|  | Intercept | 0.65 | 0.44 | 0.95 | -0.43 | 0.19 | -2.29 | .027 |
|  | Intensity | 0.83 | 0.56 | 1.23 | -0.18 | 0.20 | -0.90 | .367 |
| Unhealthy vs. mixed | |  |  |  |  |  |  |  |
|  | Intercept | 0.57 | 0.40 | 0.82 | -0.56 | 0.18 | -3.12 | .003 |
|  | Duration | 1.08 | 0.89 | 1.31 | 0.07 | 0.10 | 0.75 | .455 |
| Healthy vs. mixed | |  |  |  |  |  |  |  |
|  | Intercept | 0.65 | 0.44 | 0.96 | -0.43 | 0.19 | -2.24 | .030 |
|  | Duration | 1.18 | 0.91 | 1.54 | 0.17 | 0.13 | 1.25 | .212 |
| Unhealthy vs. mixed | |  |  |  |  |  |  |  |
|  | Intercept | 0.51 | 0.23 | 1.16 | -0.67 | 0.41 | -1.65 | .107 |
|  | Cardio vs. combination | 1.15 | 0.50 | 2.67 | 0.14 | 0.43 | 0.34 | .737 |
|  | Balance vs. combination | 2.07 | 0.51 | 8.43 | 0.73 | 0.71 | 1.02 | .310 |
|  | Strength vs. combination | 0.58 | 0.18 | 1.94 | -0.54 | 0.61 | -0.88 | .378 |
|  | Sport vs. combination | 2.55 | 0.72 | 8.95 | 0.93 | 0.64 | 1.46 | .145 |
| Healthy vs. mixed | |  |  |  |  |  |  |  |
|  | Intercept | 0.59 | 0.30 | 1.14 | -0.53 | 0.33 | -1.62 | .111 |
|  | Cardio vs. combination | 1.18 | 0.63 | 2.22 | 0.17 | 0.32 | 0.52 | .660 |
|  | Balance vs. combination | 1.23 | 0.32 | 5.06 | 0.24 | 0.70 | 0.34 | .733 |
|  | Strength vs. combination | 0.93 | 0.33 | 2.63 | -0.07 | 0.53 | -0.14 | .889 |
|  | Sport vs. combination | 1.40 | 0.40 | 4.87 | 0.34 | 0.63 | 0.53 | .596 |
| *Note*. Meal healthiness was a multicategorical outcome variable with three categories (unhealthy, mixed, healthy), generating two comparisons against mixed, the reference category: (1) Unhealthy vs. mixed meals and (2) Healthy vs. mixed meals. Exercise intensity, duration and type were tested as individual predictors of post-exercise meal healthiness. Exercise intensity and duration were treated as continuous predictors and were grand-mean centered. Exercise type was a categorical predictor with five categories (cardio, balance, strength, sport, and combination of the above). Four dummy-coded variables (with combination as the reference category) were included to examine exercise type as a predictor. | | | | | | | | |

S1 Fig. Classification of Post-Exercise and Non-Exercise Day Random Meals by Healthiness and Size

|  |
| --- |
| Note. Portion size was based on self-report ratings of the size of the meal on a scale from 1= small to 5 = big. For simplicity, on the above graphs, categories 1 = small and 2 = somewhat small have been collapsed to form “small” and categories 4 = somewhat big and 5 = big have been collapsed to form “big”. Healthiness was based on the objective coded data. |

S4 Table: Contextual Predictors of Post-Exercise Meal Healthiness (Study 2)

|  | | Odds ratio | Lower limit | Upper limit | *b* | *SE* | *t* | *p* |
| --- | --- | --- | --- | --- | --- | --- | --- | --- |
| Unhealthy vs. mixed | |  |  |  |  |  |  |  |
|  | Intercept^a^ | 0.59 | 0.28 | 1.24 | -0.52 | 0.37 | -1.42 | .162 |
|  | Feeling hungry | 0.93 | 0.48 | 1.80 | -0.07 | 0.33 | -0.20 | .839 |
| Healthy vs. mixed | |  |  |  |  |  |  |  |
|  | Intercept^a^ | 0.60 | 0.30 | 1.23 | -0.50 | 0.35 | -1.43 | .160 |
|  | Feeling hungry | 1.06 | 0.53 | 2.14 | 0.06 | 0.36 | 0.16 | .870 |
| Unhealthy vs. mixed | |  |  |  |  |  |  |  |
|  | Intercept | 0.50 | 0.34 | 0.74 | -0.69 | 0.20 | -3.52 | < .001 |
|  | Feeling stressed | 1.68 | 0.95 | 2.96 | 0.52 | 0.29 | 1.80 | .073 |
| Healthy vs. mixed | |  |  |  |  |  |  |  |
|  | Intercept | 0.65 | 0.47 | 0.90 | -0.43 | 0.16 | -2.66 | .011 |
|  | Feeling stressed | 0.86 | 0.48 | 1.53 | -0.15 | 0.30 | -0.52 | .602 |
| Unhealthy vs. mixed | |  |  |  |  |  |  |  |
|  | Intercept | 0.56 | 0.38 | 0.83 | -0.57 | 0.19 | -2.97 | .005 |
|  | In a bad mood | 1.01 | 0.43 | 2.37 | 0.01 | 0.43 | 0.03 | .978 |
| Healthy vs. mixed | |  |  |  |  |  |  |  |
|  | Intercept | 0.65 | 0.47 | 0.89 | -0.43 | 0.16 | -2.73 | .009 |
|  | In a bad mood | 0.71 | 0.38 | 1.34 | -0.34 | 0.32 | -1.06 | .292 |
| Unhealthy vs. mixed | |  |  |  |  |  |  |  |
|  | Intercept | 0.52 | 0.33 | 0.81 | -0.66 | 0.22 | -2.98 | .005 |
|  | Feeling tired | 1.20 | 0.72 | 2.03 | 0.19 | 0.26 | 0.70 | .482 |
| Healthy vs. mixed | |  |  |  |  |  |  |  |
|  | Intercept | 0.63 | 0.42 | 0.95 | -0.46 | 0.21 | -2.24 | .030 |
|  | Feeling tired | 1.01 | 0.60 | 1.68 | 0.01 | 0.26 | 0.03 | .979 |
| Unhealthy vs. mixed | |  |  |  |  |  |  |  |
|  | Intercept | 0.60 | 0.40 | 0.89 | -0.52 | 0.20 | -2.59 | .013 |
|  | No other food options | 0.75 | 0.45 | 1.26 | -0.29 | 0.26 | -1.10 | .272 |
| Healthy vs. mixed | |  |  |  |  |  |  |  |
|  | Intercept | 0.64 | 0.44 | 0.92 | -0.45 | 0.18 | -2.50 | .016 |
|  | No other food options | 0.98 | 0.48 | 1.98 | -0.02 | 0.36 | -0.06 | .950 |
| Unhealthy vs. mixed | |  |  |  |  |  |  |  |
|  | Intercept | 0.77 | 0.53 | 1.13 | -0.26 | 0.19 | -1.37 | .176 |
|  | Planned to eat that food | **0.51** | **0.29** | **0.88** | **-0.68** | **0.28** | **-2.41** | **.016** |
| Healthy vs. mixed | |  |  |  |  |  |  |  |
|  | Intercept | 0.54 | 0.37 | 0.80 | -0.61 | 0.19 | -3.16 | .003 |
|  | Planned to eat that food | 1.30 | 0.75 | 2.28 | 0.27 | 0.28 | 0.94 | .350 |
| Unhealthy vs. mixed | |  |  |  |  |  |  |  |
|  | Intercept | 0.42 | 0.21 | 0.81 | -0.87 | 0.33 | -2.63 | .011 |
|  | Food readily available | 1.46 | 0.69 | 3.07 | 0.38 | 0.38 | 0.99 | .323 |
| Healthy vs. mixed | |  |  |  |  |  |  |  |
|  | Intercept | 0.45 | 0.25 | 0.82 | -0.80 | 0.30 | -2.67 | .010 |
|  | Food readily available | 1.53 | 0.76 | 3.09 | 0.43 | 0.36 | 1.19 | .233 |
| Unhealthy vs. mixed | |  |  |  |  |  |  |  |
|  | Intercept | 0.38 | 0.24 | 0.61 | -0.96 | 0.23 | -4.16 | < .001 |
|  | Cravings for food | **2.54** | **1.47** | **4.41** | **0.93** | **0.28** | **3.33** | **< .001** |
| Healthy vs. mixed | |  |  |  |  |  |  |  |
|  | Intercept | 0.72 | 0.50 | 1.05 | -0.33 | 0.19 | -1.76 | .085 |
|  | Cravings for food | 0.61 | 0.33 | 1.13 | -0.49 | 0.31 | -1.58 | .116 |
|  |  |  |  |  |  |  |  |  |
| Unhealthy vs. mixed | |  |  |  |  |  |  |  |
|  | Intercept | 0.64 | 0.41 | 0.99 | -0.45 | 0.22 | -2.04 | .047 |
|  | Eating alone | 0.77 | 0.41 | 1.42 | -0.27 | 0.31 | -0.85 | .396 |
| Healthy vs. mixed | |  |  |  |  |  |  |  |
|  | Intercept | 0.61 | 0.44 | 0.85 | -0.49 | 0.16 | -3.02 | .004 |
|  | Eating alone | 1.07 | 0.61 | 1.89 | 0.07 | 0.29 | 0.25 | .802 |
| Unhealthy vs. mixed | |  |  |  |  |  |  |  |
|  | Intercept | 0.58 | 0.39 | 0.86 | -0.55 | 0.20 | -2.78 | .008 |
|  | In a rush | 0.87 | 0.44 | 1.72 | -0.14 | 0.35 | -0.41 | .681 |
| Healthy vs. mixed | |  |  |  |  |  |  |  |
|  | Intercept | 0.64 | 0.46 | 0.89 | -0.45 | 0.17 | -2.72 | .009 |
|  | In a rush | 0.96 | 0.50 | 1.86 | -0.04 | 0.34 | -0.12 | .902 |
| Unhealthy vs. mixed | |  |  |  |  |  |  |  |
|  | Intercept | 0.74 | 0.45 | 1.21 | -0.30 | 0.25 | -1.22 | .228 |
|  | Eating at home | 0.65 | 0.37 | 1.14 | -0.44 | 0.29 | -1.51 | .133 |
| Healthy vs. mixed | |  |  |  |  |  |  |  |
|  | Intercept | 0.74 | 0.48 | 1.14 | -0.30 | 0.22 | -1.39 | .170 |
|  | Eating at home | 0.79 | 0.46 | 1.37 | -0.23 | 0.28 | -0.84 | .401 |
| *Note.* Meal healthiness was a multicategorical outcome variable with three categories (unhealthy, mixed, healthy), generating two comparisons against mixed, the reference category: (1) Unhealthy vs. mixed meals and (2) Healthy vs. mixed meals. Each of the 11 contextual factors are dichotomous predictors (1 = yes, 0 = no). They were examined as individual predictors. Bold denotes that the predictor was significant.  ^a^Intercept of the hierarchical generalized linear model (HGLM) refers to when the value of the contextual predictor (i.e., feeling hungry) = 0 (i.e., not hungry). | | | | | | | | |

S5 Table: Contextual Predictors of Non-exercise Day Meal Healthiness (Study 2)

|  | | Odds ratio | Lower limit | Upper limit | *b* | *SE* | *t* | *p* |
| --- | --- | --- | --- | --- | --- | --- | --- | --- |
| Unhealthy vs. mixed | |  |  |  |  |  |  |  |
|  | Intercept^a^ | 0.91 | 0.63 | 1.32 | -0.09 | 0.19 | -0.50 | .616 |
|  | Feeling hungry | 1.13 | 0.76 | 1.67 | 0.12 | 0.20 | 0.60 | .522 |
| Healthy vs. mixed | |  |  |  |  |  |  |  |
|  | Intercept^a^ | 0.85 | 0.61 | 1.17 | -0.17 | 0.16 | -1.05 | .299 |
|  | Feeling hungry | 0.98 | 0.71 | 1.36 | -0.02 | 0.16 | -0.12 | .903 |
| Unhealthy vs. mixed | |  |  |  |  |  |  |  |
|  | Intercept | 0.86 | 0.66 | 1.11 | -0.16 | 0.13 | -1.22 | .229 |
|  | Feeling stressed | **1.83** | **1.19** | **2.82** | **0.60** | **0.22** | **2.75** | **.006** |
| Healthy vs. mixed | |  |  |  |  |  |  |  |
|  | Intercept | 0.80 | 0.63 | 1.00 | -0.23 | 0.11 | -1.97 | .054 |
|  | Feeling stressed | 1.25 | 0.85 | 1.84 | 0.22 | 0.20 | 1.11 | .267 |
| Unhealthy vs. mixed | |  |  |  |  |  |  |  |
|  | Intercept | 0.94 | 0.74 | 1.20 | -0.06 | 0.12 | -0.50 | .620 |
|  | In a bad mood | 1.52 | 0.82 | 2.82 | 0.42 | 0.32 | 1.32 | .188 |
| Healthy vs. mixed | |  |  |  |  |  |  |  |
|  | Intercept | 0.82 | 0.65 | 1.05 | -0.19 | 0.11 | -1.62 | .111 |
|  | In a bad mood | 1.15 | 0.512 | 2.56 | 0.14 | 0.41 | 0.33 | .740 |
| Unhealthy vs. mixed | |  |  |  |  |  |  |  |
|  | Intercept | 0.97 | 0.76 | 1.26 | -0.03 | 0.13 | -0.21 | .835 |
|  | Feeling tired | 1.03 | 0.67 | 1.58 | 0.03 | 0.22 | 0.23 | .903 |
| Healthy vs. mixed | |  |  |  |  |  |  |  |
|  | Intercept | 0.94 | 0072 | 1.21 | -0.07 | 0.13 | -0.51 | .610 |
|  | Feeling tired | 0.70 | 0.44 | 1.11 | -0.36 | 0.24 | -1.51 | .133 |
| Unhealthy vs. mixed | |  |  |  |  |  |  |  |
|  | Intercept | 1.09 | 0.85 | 1.41 | 0.09 | 0.13 | 0.70 | .487 |
|  | No other food options | **0.58** | **0.38** | **0.91** | **-0.54** | **0.22** | **-2.40** | **.016** |
| Healthy vs. mixed | |  |  |  |  |  |  |  |
|  | Intercept | 0.85 | 0.66 | 1.09 | -0.16 | 0.13 | -1.30 | .198 |
|  | No other food options | 0.93 | 0.63 | 1.36 | -0.08 | 0.20 | -0.39 | .700 |
| Unhealthy vs. mixed | |  |  |  |  |  |  |  |
|  | Intercept | 1.44 | 1.08 | 1.93 | 0.37 | 0.15 | 2.52 | .015 |
|  | Planned to eat that food | **0.38** | **0.27** | **0.55** | **-0.96** | **0.18** | **-5.25** | **< .001** |
| Healthy vs. mixed | |  |  |  |  |  |  |  |
|  | Intercept | 0.92 | 0.69 | 1.24 | -0.08 | 0.15 | -0.53 | .598 |
|  | Planned to eat that food | 0.81 | 0.57 | 1.16 | -0.21 | 0.18 | -1.14 | .255 |
| Unhealthy vs. mixed | |  |  |  |  |  |  |  |
|  | Intercept | 1.08 | 0.73 | 1.60 | 0.08 | 0.20 | 0.38 | .702 |
|  | Food readily available | 0.88 | 0.59 | 1.33 | -0.13 | 0.21 | -0.61 | .546 |
| Healthy vs. mixed | |  |  |  |  |  |  |  |
|  | Intercept | 0.87 | 0.60 | 1.25 | -0.14 | 0.18 | -0.79 | .433 |
|  | Food readily available | 0.95 | 0.63 | 1.43 | -0.05 | 0.21 | -0.25 | .806 |
| Unhealthy vs. mixed | |  |  |  |  |  |  |  |
|  | Intercept | 0.73 | 0.54 | 0.97 | -0.31 | 0.15 | -2.10 | .040 |
|  | Cravings for food | **2.19** | **1.43** | **3.34** | **0.78** | **0.22** | **3.62** | **< .001** |
| Healthy vs. mixed | |  |  |  |  |  |  |  |
|  | Intercept | 1.02 | 0.78 | 1.32 | 0.02 | 0.13 | 0.12 | .908 |
|  | Cravings for food | **0.41** | **0.28** | **0.62** | **-0.88** | **0.21** | **-4.26** | **< .001** |
|  |  |  |  |  |  |  |  |  |
| Unhealthy vs. mixed | |  |  |  |  |  |  |  |
|  | Intercept | 1.00 | 0.76 | 1.32 | < 0.01 | 0.14 | 0.02 | .985 |
|  | Eating alone | 0.96 | 0.68 | 1.34 | -0.04 | 0.17 | -0.26 | .796 |
| Healthy vs. mixed | |  |  |  |  |  |  |  |
|  | Intercept | 0.77 | 0.57 | 1.04 | -0.27 | 0.15 | -1.75 | .085 |
|  | Eating alone | 1.19 | 0.82 | 1.73 | 0.17 | 0.19 | 0.89 | .372 |
| Unhealthy vs. mixed | |  |  |  |  |  |  |  |
|  | Intercept | 1.01 | 0.79 | 1.31 | 0.01 | 0.13 | 0.12 | .907 |
|  | In a rush | 0.78 | 0.45 | 1.37 | -0.25 | 0.29 | -0.86 | .390 |
| Healthy vs. mixed | |  |  |  |  |  |  |  |
|  | Intercept | 0.84 | 0.66 | 1.07 | -0.17 | 0.12 | -1.48 | .146 |
|  | In a rush | 0.96 | 0.60 | 1.55 | -0.04 | 0.24 | -0.16 | .875 |
| Unhealthy vs. mixed | |  |  |  |  |  |  |  |
|  | Intercept | 1.18 | 0.86 | 1.64 | 0.17 | 0.16 | 1.04 | .301 |
|  | Eating at home | 0.73 | 0.48 | 1.10 | -0.31 | 0.21 | -1.50 | .135 |
| Healthy vs. mixed | |  |  |  |  |  |  |  |
|  | Intercept | 0.68 | 0.49 | 0.94 | -0.39 | 0.16 | -2.41 | .020 |
|  | Eating at home | 1.36 | 0.95 | 1.96 | 0.31 | 0.18 | 1.67 | .095 |
| *Note.* Meal healthiness was a multicategorical outcome variable with three categories (unhealthy, mixed, healthy), generating two comparisons against mixed, the reference category: (1) Unhealthy vs. mixed meals and (2) Healthy vs. mixed meals. Each of the 11 contextual factors are dichotomous predictors (1 = yes, 0 = no). They were examined as individual predictors. Bold denotes that the predictor was significant.  ^a^Intercept of the hierarchical generalized linear model (HGLM) refers to when the value of the contextual predictor (i.e., feeling hungry) = 0 (i.e., not hungry). | | | | | | | | |

S6 Table: Characteristics of Exercise as Predictors of Post-Exercise Meal Healthiness (Study 2)

|  | | Odds ratio | Lower limit | Upper limit | *b* | *SE* | *t* | *p* |
| --- | --- | --- | --- | --- | --- | --- | --- | --- |
| Unhealthy vs. mixed | |  |  |  |  |  |  |  |
|  | Intercept | 0.56 | 0.39 | 0.83 | -0.57 | 0.19 | -3.03 | .004 |
|  | Intensity | 1.02 | 0.72 | 1.44 | 0.02 | 0.18 | 0.10 | .919 |
| Healthy vs. mixed | |  |  |  |  |  |  |  |
|  | Intercept | 0.63 | 0.46 | 0.86 | -0.47 | 0.16 | -3.00 | .004 |
|  | Intensity | 0.85 | 0.60 | 1.21 | -0.16 | 0.18 | -0.91 | .365 |
| Unhealthy vs. mixed | |  |  |  |  |  |  |  |
|  | Intercept | 0.56 | 0.39 | 0.82 | -0.57 | 0.19 | -3.03 | .004 |
|  | Duration | 1.01 | 0.82 | 1.23 | 0.01 | 0.10 | 0.05 | .958 |
| Healthy vs. mixed | |  |  |  |  |  |  |  |
|  | Intercept | 0.63 | 0.47 | 0.86 | -0.46 | 0.15 | -3.00 | .004 |
|  | Duration | 1.02 | 0.85 | 1.22 | 0.02 | 0.09 | 0.22 | .823 |
| Unhealthy vs. mixed | |  |  |  |  |  |  |  |
|  | Intercept | 0.90 | 0.39 | 2.09 | -0.11 | 0.42 | -0.26 | .799 |
|  | Cardio vs. combination | 0.74 | 0.33 | 1.67 | -0.30 | 0.41 | -0.73 | .467 |
|  | Balance vs. combination | **0.17** | **0.04** | **0.76** | **-1.76** | **0.76** | **-2.33** | **.021** |
|  | Strength vs. combination | 0.56 | 0.19 | 1.62 | -0.58 | 0.54 | -1.08 | .282 |
|  | Sport vs. combination | 0.48 | 0.15 | 1.59 | -0.73 | 0.61 | -1.21 | .228 |
| Healthy vs. mixed | |  |  |  |  |  |  |  |
|  | Intercept | 0.68 | 0.29 | 1.59 | -0.39 | 0.42 | -0.92 | .365 |
|  | Cardio vs. combination | 0.86 | 0.37 | 1.98 | -0.16 | 0.43 | -0.37 | .716 |
|  | Balance vs. combination | 1.11 | 0.41 | 3.05 | 0.11 | 0.51 | 0.21 | .833 |
|  | Strength vs. combination | 1.11 | 0.42 | 2.94 | 0.11 | 0.49 | 0.21 | .831 |
|  | Sport vs. combination | 0.72 | 0.25 | 2.11 | -0.33 | 0.55 | -0.60 | .550 |
| *Note*. Meal healthiness was a multicategorical outcome variable with three categories (unhealthy, mixed, healthy), generating two comparisons against mixed, the reference category: (1) Unhealthy vs. mixed meals and (2) Healthy vs. mixed meals. Exercise intensity, duration and type were tested as individual predictors of post-exercise meal healthiness. Exercise intensity and duration were treated as continuous predictors and were grand-mean centered. Exercise type was a categorical predictor with five categories (cardio, balance, strength, sport, and combination of the above). Four dummy-coded variables (with combination as the reference category) were included to examine exercise type as a predictor. Bold denotes that the predictor was significant. | | | | | | | | |

S7 Table: Contextual Predictors of Post-Exercise Meal Portion Size

|  | *b* | *SE* | *t* | *p* |
| --- | --- | --- | --- | --- |
| Intercept^a^ | 2.66 | 0.12 | 22.19 | < .001 |
| Feeling hungry | **0.53** | **0.10** | **5.30** | **< .001** |
| Intercept | 3.06 | 0.08 | 36.77 | < .001 |
| Feeling stressed | 0.02 | 0.15 | 0.15 | .884 |
| Intercept | 3.05 | 0.08 | 38.01 | < .001 |
| In a bad mood | 0.10 | 0.23 | 0.45 | .656 |
| Intercept | 3.06 | 0.09 | 35.05 | < .001 |
| Feeling tired | 0.01 | 0.09 | 0.06 | .954 |
| Intercept | 3.05 | 0.09 | 32.23 | < .001 |
| No other food options | 0.04 | 0.15 | 0.27 | .787 |
| Intercept | 3.09 | 0.10 | 29.63 | < .001 |
| Planned to eat that food | -0.06 | 0.12 | -0.51 | .607 |
| Intercept | 3.39 | 0.14 | 24.32 | < .001 |
| Food was readily available | **-0.42** | **0.13** | **-3.23** | **.001** |
| Intercept | 3.02 | 0.09 | 33.35 | < .001 |
| Cravings for food | 0.12 | 0.09 | 1.35 | .178 |
| Intercept | 3.27 | 0.10 | 32.15 | < .001 |
| Eating alone | **-0.43** | **0.10** | **-4.46** | **< .001** |
| Intercept | 3.07 | 0.09 | 35.26 | < .001 |
| In a rush | -0.05 | 0.18 | -0.28 | .783 |
| Intercept | 3.14 | 0.10 | 30.05 | < .001 |
| Eating at home | -0.12 | 0.11 | -1.10 | .271 |
| *Note.* Self-reported portion size was treated as a continuous outcome (1 = *small*, 5 = *big*). Each of the 11 contextual factors are dichotomous predictors (1 = yes, 0 = no). They were examined as individual predictors. Bold denotes that the predictor was significant.  ^a^Intercept refers to when the value of the contextual predictor (i.e., feeling hungry) = 0 (i.e., not hungry). | | | | |

S8 Table: Contextual Predictors of Non-exercise Day Meal Portion Size

|  | *b* | *SE* | *t* | *p* |
| --- | --- | --- | --- | --- |
| Intercept^a^ | 2.63 | 0.09 | 27.74 | < .001 |
| Feeling hungry | **0.35** | **0.09** | **3.91** | **< .001** |
| Intercept | 2.87 | 0.07 | 42.77 | < .001 |
| Feeling stressed | -0.06 | 0.09 | -0.62 | .533 |
| Intercept | 2.83 | 0.06 | 44.19 | < .001 |
| In a bad mood | 0.26 | 0.15 | 1.80 | .073 |
| Intercept | 2.78 | 0.06 | 45.31 | < .001 |
| Feeling tired | **0.21** | **0.08** | **2.65** | **.008** |
| Intercept | 2.85 | 0.07 | 41.01 | < .001 |
| No other food options | 0.01 | 0.10 | 0.09 | .932 |
| Intercept | 2.72 | 0.08 | 35.68 | < .001 |
| Planned to eat that food | **0.31** | **0.09** | **3.43** | **< .001** |
| Intercept | 3.21 | 0.08 | 38.06 | < .001 |
| Food was readily available | **-0.49** | **0.09** | **-5.54** | **< .001** |
| Intercept | 2.80 | 0.07 | 39.62 | < .001 |
| Cravings for food | 0.17 | 0.09 | 1.83 | .068 |
| Intercept | 2.99 | 0.08 | 36.99 | < .001 |
| Eating alone | **-0.28** | **0.11** | **-2.67** | **.008** |
| Intercept | 2.89 | 0.07 | 44.20 | < .001 |
| In a rush | -0.23 | 0.12 | -1.97 | .050 |
| Intercept | 2.97 | 0.10 | 30.81 | < .001 |
| Eating at home | -0.18 | 0.09 | -1.92 | .055 |
| *Note.* Self-reported portion size was treated as a continuous outcome (1 = *small*, 5 = *big*). Each of the 11 contextual factors are dichotomous predictors (1 = yes, 0 = no). They were examined as individual predictors. Bold denotes that the predictor was significant.  ^a^Intercept refers to when the value of the contextual predictor (i.e., feeling hungry) = 0 (i.e., not hungry). | | | | |

S9 Table: Characteristics of Exercise as Predictors of Post-Exercise Meal Portion Size

|  | *b* | *SE* | *t* | *p* |
| --- | --- | --- | --- | --- |
| Intercept | 3.18 | 0.12 | 26.59 | < .001 |
| Intensity | 0.23 | 0.13 | 1.84 | .072 |
| Intercept | 3.20 | 0.12 | 26.48 | < .001 |
| Duration | 0.11 | 0.08 | 1.48 | .144 |
| Intercept | 2.97 | 0.42 | 7.01 | < .001 |
| Cardio vs. combination | 0.17 | 0.48 | 0.36 | .720 |
| Balance vs. combination | 0.41 | 0.46 | 0.88 | .381 |
| Strength vs. combination | 0.29 | 0.49 | 0.60 | .552 |
| Sport vs. combination | 0.68 | 0.50 | 1.37 | .179 |
| *Note*. Self-reported portion size was treated as a continuous outcome (1 = *small*, 5 = *big*). Exercise intensity, duration and type were tested as individual predictors of post-exercise meal portion size. Exercise intensity and duration were treated as continuous predictors and were grand-mean centered. Exercise type was a categorical predictor with five categories (cardio, balance, strength, sport, and combination of the above). Four dummy-coded variables (with combination as the reference category) were included to examine exercise type as a predictor. | | | | |
